# Generating dynamical neuroimaging spatiotemporal representations (DyNeuSR) using topological data analysis

**DOI:** 10.1162/netn_a_00093

**Published:** 2019-07-01

**Authors:** Caleb Geniesse, Olaf Sporns, Giovanni Petri, Manish Saggar

**Affiliations:** Biophysics Program, Stanford University, Stanford, CA, USA; Department of Psychiatry and Behavioral Sciences, Stanford University, Stanford, CA, USA; Department of Psychological and Brain Sciences, Indiana University, Bloomington, IN, USA; ISI Foundation, Turin, Italy; ISI Global Science Foundation, New York, NY, USA; Biophysics Program, Stanford University, Stanford, CA, USA; Department of Psychiatry and Behavioral Sciences, Stanford University, Stanford, CA, USA

**Keywords:** Brain dynamics, TDA, fMRI, Brain networks, Mapper

## Abstract

In this article, we present an open source neuroinformatics platform for exploring, analyzing, and validating distilled graphical representations of high-dimensional neuroimaging data extracted using topological data analysis (TDA). TDA techniques like Mapper have been recently applied to examine the brain’s dynamical organization during ongoing cognition without averaging data in space, in time, or across participants at the outset. Such TDA-based approaches mark an important deviation from standard neuroimaging analyses by distilling complex high-dimensional neuroimaging data into simple—yet neurophysiologically valid and behaviorally relevant—representations that can be interactively explored at the single-participant level. To facilitate wider use of such techniques within neuroimaging and general neuroscience communities, our work provides several tools for visualizing, interacting with, and grounding TDA-generated graphical representations in neurophysiology. Through Python-based Jupyter notebooks and open datasets, we provide a platform to assess and visualize different intermittent stages of Mapper and examine the influence of Mapper parameters on the generated representations. We hope this platform could enable researchers and clinicians alike to explore topological representations of neuroimaging data and generate biological insights underlying complex mental disorders.

## Author Summary

To develop biologically grounded psychiatric diagnosis, researchers and clinicians need tools for distilling complex high-dimensional neuroimaging data into simple yet interactive and clinically relevant representations. Further, for translational outcomes, these representations should be conceivable at the single-participant level. Topological data analysis techniques such as Mapper allow generation of these representations. Here, we introduce a set of tools that can facilitate wider acceptance of Mapper within the neuroscience community and provide a series of easy-to-follow steps for visualizing Mapper-generated graphical representations. We provide detailed examples to reveal the “under-the-hood” workings of Mapper.

## INTRODUCTION

Capturing and quantifying dynamic fluctuations in neuronal activity is critical for understanding how the brain dynamically reorganizes during ongoing cognition. Although current neuroimaging technologies enable us to measure brain function at very high spatiotemporal resolutions, most traditional approaches to neuroimaging data analysis invariably collapse high-resolution neuroimaging data across spatiotemporal scales at the outset (Preti, Bolton, & Van De Ville, 2016). The resulting loss of spatiotemporal precision impedes the interpretation of such high-dimensional neuroimaging data and may obscure important detail related to temporal dynamics and individual differences. For better translational outcomes, there is a need for interactive data-driven methods for analyzing and visualizing neuroimaging data that are capable of delivering behaviorally relevant insights at the single-participant level without collapsing spatiotemporal data at the outset.

Several innovative methods have been proposed to examine and quantify fluctuations in both functional activity (Karahanoğlu & Van De Ville, 2015; Liu & Duyn, 2013; Liu, Zhang, Chang, & Duyn, 2018) and connectivity (Preti et al., 2016; Shine et al., 2015; Xu & Lindquist, 2015). These approaches provide valuable insights; however, they cannot uncover the threshold-free optimal spatiotemporal scale that best captures behaviorally relevant dynamics (Preti et al., 2016). More recently, an approach based on topological data analysis (TDA) called Mapper (Carlsson, 2009; Lum et al., 2013; Singh, Mémoli & Carlsson, 2007) has been used to graphically represent the brain’s overall dynamical organization (i.e., the shape graph) without arbitrarily collapsing data in space or time (Saggar et al., 2018). Intuitively, Mapper helps construct a skeletonized graph of a high-dimensional dataset to encapsulate the original shape of the data by representing similar points as more closely linked than dissimilar points in the generated shape graph. For example, in case of studying anatomical heterogeneity across participants, the data points could be individual participants themselves and the Mapper-generated graph would link participants with similar anatomical features closer as compared with participants with dissimilar anatomy. Mapper-generated representation is analogous to generating a subway map that can capture the essential features of a system while potentially reducing the effect of noisy data. Further, the generated representations can be interactively visualized, quantified in a variety of ways using graph theory, validated by anchoring them to brain anatomy, and constructed at the level of individual participants or populations, making them suitable for exploratory and translational research purposes.

Although several different Mapper software allow construction of such shape graphs for any kind of data that can be represented as a matrix (including neuroimaging data), to our knowledge, none of these tools were designed with explicit built-in support for visualization and analysis of those graphs in the context of neuroscience (see Table 1). Moreover, while software packages have been developed specifically for neuroimaging data analysis and visualization, none of these tools were designed specifically to work with shape graphs produced by Mapper. As such, integrating Mapper with existing neuroimaging tools into a full data analysis pipeline poses a steep learning curve and a significant challenge for many neuroscience researchers. To overcome these hurdles, we believe researchers need tools that connect Mapper with existing neuroimaging software.

**Table 1.** A survey of available Mapper software.

**Software**	**Programming language**	**License**	**Built-in support for neuroscience**	**Link**
Ayasdi	Python	Proprietary	No	ayasdi.com
KeplerMapper	Python	MIT	No	github.com/MLWave/kepler-mapper
Python Mapper	Python	GPL	No	danifold.net/mapper
TDAmapper	R	GPL	No	github.com/paultpearson/TDAmapper
MapperTools	Python	GPL	No	github.com/alpatania/MapperTools

**Figure 1.**
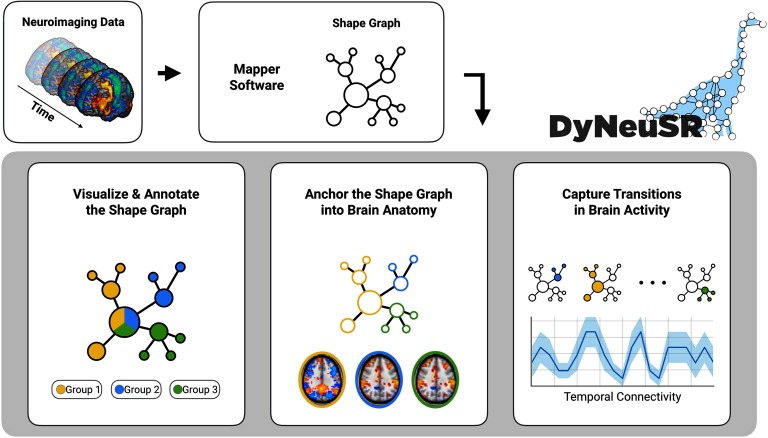
Overview of DyNeuSR. Once the Mapper graph is generated using any of the existing tools listed in Table 1, DyNeuSR allows for annotating using meta information, anchoring graph nodes in to neurophysiology, and capturing temporal (or over samples) variations.

In light of this need, we developed DyNeuSR, an open source platform for exploring, analyzing, and validating topological properties and neurophysiological correlates of Mapper-generated graphs (see Figure 1). The DyNeuSR toolkit and source code are available at our website (Geniesse, Sporns, Petri, & Saggar, 2018).

In this paper, we provide some examples to illustrate the key features of DyNeuSR. First, we introduce Mapper and its workings using a synthetic 3-D trefoil knot dataset. Using the trefoil dataset, we also compare Mapper with other traditional dimensionality reduction techniques. Second, we introduce the features of DyNeuSR using a real neuroimaging dataset (Haxby et al., 2001). Specifically, we show how to (a) visualize and annotate shape graphs with meta-information; (b) anchor shape graphs into brain anatomy and physiology; and (c) quantify shape graphs to capture transitions in brain activity. Together, these demonstrations highlight how DyNeuSR provides a simple interface between Mapper and neuroimaging data analysis and visualization.

## RESULTS

The results are presented in two parts. First, we use DyNeuSR to introduce, visualize, and motivate Mapper on a synthetic 3-D trefoil knot. Second, we use DyNeuSR to analyze and interpret shape graphs generated from a real neuroimaging dataset. To view the respective code and results, please refer to the Jupyter notebook provided in Supplemental Data 1 and 2 (Supporting Information).

### Introducing and Visualizing Mapper

Formally, Mapper produces the topological skeleton of a dataset by combining together the dataset and a map defined on it (Carlsson, 2009; Lum et al., 2013; Singh et al., 2007). With these two elements, it computes a cover of the codomain of the map, which is the actual skeleton. In practice, this process can be summarized in four stages: (a) slicing the data; (b) binning data points according to slices from the first stage; (c) clustering data points within each bin; and (d) linking the clusters that share points across bins.

**Figure 2.**
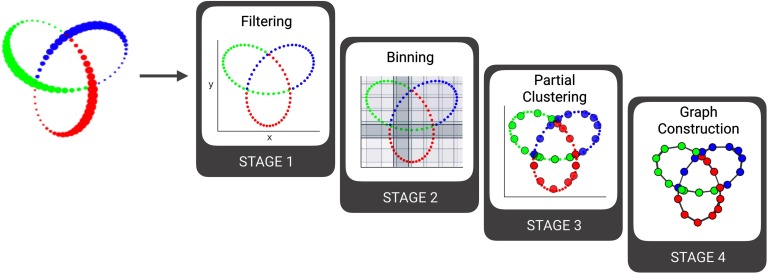
Visualizing the Stages of Mapper. This figure shows the results from a visual inspection of Mapper intermediates for synthetic data sampled from a 3-D trefoil knot.

To better understand these stages, DyNeuSR provides tools for visualizing how data are transformed at each stage. See Figure 2 for a visual representation of each stage. In the following section, we discuss how the trefoil knot data are transformed during each of the four stages of Mapper. We then highlight the advantages of Mapper as compared with standard dimensionality reduction methods.

#### Stages of Mapper.

High-dimensional data are transformed into a lower dimensional graphical representation as it passes through the four stages of the Mapper algorithm. The first stage of Mapper involves filtering high-dimensional data into a lower dimension. Note that this filtering step is not equivalent to standard dimensionality reduction; instead, the filters in Mapper are used as lenses to slice the data. The shape of the data captured in the later stages and final output of Mapper often depend on how the data were sliced, and thus, the choice of filter function and Mapper parameters is critical (see the Discussion section). The ability to slice data in several ways, however, makes this tool extremely versatile for data exploration and feature extraction (Phinyomark, Ibanez-Marcelo, & Petri, 2017). For the 3-D trefoil knot data, filtering along any subset of the three dimensions will produce a simple lens that is easy to visualize (see Figure 2). The shape graph shown in Figure 2 was generated using the first and second dimensions of the 3-D trefoil knot data as a lens; however, other pairs of dimensions produce similar results (see Supplemental Figure 1A; Supporting Information).

**Figure 3.**
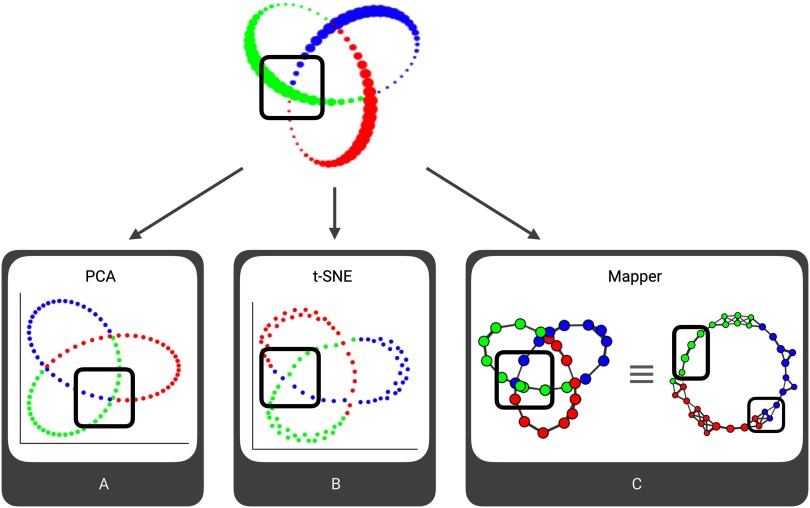
Visualizing the advantages of Mapper. This figure compares representations of the synthetical 3-D trefoil knot generated using traditional dimensionality reduction techniques, including linear (e.g., PCA) and nonlinear (e.g., t-SNE) approaches with one generated using Mapper. A subset of the data points can be mapped from the high-dimensional space (top) to each of the low-dimensional representations (bottom). Note, blue and green points within the subset of data are separated by the third dimension in the high-dimensional space. PCA (A) and t-SNE (B) both fail to resolve this separation—the blue and green points are positioned similarly in the reduced-dimension space. In contrast, Mapper (C) represents these points as two different unconnected nodes in the shape graphs. Since Mapper performs clustering on the original three-dimensional data, the blue and green points are assigned to different clusters because of the separation in the third dimension. Further, the same shape graph is shown in two different layouts, revealing that the result is in fact a circle. For additional results showing how Mapper recovers an actual topological circle, see Supplemental Figure 1 (Supporting Information).

After filtering the data using a lens, the data are sliced or binned along each filter dimension in the lens into overlapping bins, that is, a cover parameterized by resolution (R) and gain (G). Here, the resolution corresponds to the number of bins and the gain to the relative overlap between them. The shape graph of the trefoil data shown in Figures 2 and 3 was generated using a low resolution (R = 6) and a high gain equivalent to an 80% overlap between bins (G = 5). Supplemental Figure 1B (Supporting Information) provides similar results using different resolution and gain parameters.

The third stage pertains to partial clustering of data points within each bin. Any clustering method can be used, and, importantly, the clustering is performed in the original high-dimensional space. This clustering step compresses the original data by grouping similar data. Because of the overlap between bins, any two adjacent bins along each filter dimension can share data points. In the last step, adjacent bins with non-empty intersection (i.e., with common data points) are joined together into a simplified skeleton representation of the original data.

#### Advantages of Mapper.

With respect to standard dimensionality reduction techniques, Mapper is advantageous because it combines dimensionality reduction with clustering in the original high-dimensional space. Standard dimensionality reduction techniques can only project data points to a lower dimensional space, where the analysis is then performed. Thus, regardless how accurate the projection, these methods are bound to lose some of the information contained in the dataset for the sake of interpretability. Conversely, Mapper uses information from the lower dimensional lens to augment the information present in the original high-dimensional space.

The trefoil knot example is particularly illustrative. Standard dimensionality reduction techniques yield complicated overlapping patterns in two dimensions (Figure 3A and 3B), while Mapper utilizes two-dimensional projection information jointly with clustering in three dimensions to return nonoverlapping clusters at the intersection points of the knot (emphasized in Figure 3C). This allows Mapper to tease apart the real structure of the trefoil knot: a topological circle. For additional results, see Supplemental Figure 1 (Supporting Information).

### Analyzing and Interpreting Shape Graphs

In this section, we demonstrate three different ways to extract insights from shape graph representations of real brain activity data with DyNeuSR. Specifically, we explore the functional magnetic resonance imaging (fMRI) dataset from a study on face and object recognition in the human ventral temporal cortex (Haxby et al., 2001). Data were collected while visual stimuli from eight different categories (e.g., faces, cats, five categories of man-made objects, and scrambled control) were presented to six different subjects across 12 sessions (per subject). Here, we applied Mapper to the fMRI brain activity patterns of response measured in the ventral stream, which is a major pathway involved with object identification and recognition. For each of the six subjects, the input to Mapper was a matrix with time frames as rows and voxels as columns. Specifically, we used 242 time frames from Sessions 4 and 5, and voxels from each subjects’ ventral temporal cortex (e.g., 577 voxels for the first subject). KeplerMapper (Saul & van Veen, 2017) was used to generate the shape graphs. We used Stochastic Neighborhood Embedding (t-SNE; Maaten & Hinton, 2008) as a nonlinear lens for filtering, and a resolution of 17 and gain of 3 (i.e., 66.7% overlap) as cover parameters for binning. We also explored a linear lens (PCA) to examine the Haxby dataset; please see Supplemental Figure 2 (Supporting Information) for more information.

In the following subsections, we will describe three different ways that the shape graph can be related to neurophysiology using DyNeuSR. Supplemental Data 2 (Supporting Information) includes a Jupyter notebook containing the code used to download and prepare the data, generate the shape graphs using Mapper, and perform the analyses of the shape graphs using DyNeuSR.

#### Visualizing and annotating shape graphs.

The shape graphs generated by Mapper provide a mapping between data points in the original dataset and nodes in the shape graph. This mapping can also be used to annotate the shape graph using additional meta-information not used in the analysis (e.g., target variables or outcomes). DyNeuSR makes it easy to annotate and color nodes in the shape graph based on the meta-information.

To demonstrate this, we annotated nodes in each of the six subjects’ shape graphs based on the categories of stimuli presented at each time frame. For each time frame, we know the category of visual stimulus presented. Thus, every node can be assigned a set of stimulus labels corresponding to the set of time frames contained in that node. These assignments can be used for annotating (or coloring) the shape graph. To represent the relative proportion of the different stimulus labels assigned, DyNeuSR represents each node as a pie chart.

DyNeuSR provides a user interface for visualizing and interacting with the shape graph (see Supplemental Figure 4 for an example; Supporting Information). Using this interface, we were able to quickly visualize the different proportions of visual stimuli associated with the nodes in the shape graph, as well as different regions of the shape graph corresponding to differently colored nodes. With this visualization alone, researchers can glean insights into how separable (or not) different kinds of visual stimuli are based on their respectively evoked brain activation patterns.

**Figure 4.**
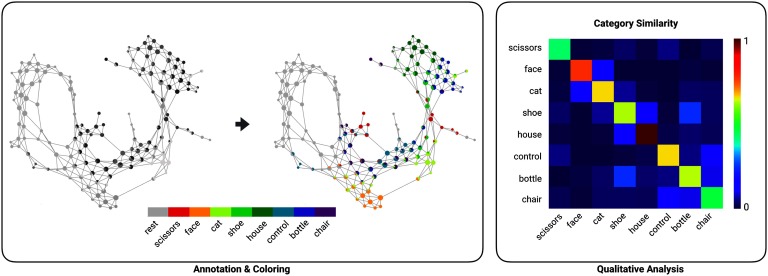
Annotating the shape graph with meta-information. The left panel shows how the shape graph can be annotated and colored with DyNeuSR. Nodes in the shape graph are represented as pie charts to help visualize the different categories of visual stimuli associated with each node (e.g., faces, houses). The annotated shape graph can be analyzed, for example, to examine category specificity. In the right panel, the similarity of categories is shown for the first subject from the Haxby dataset. This similarity can be estimated by comparing the connectivity of nodes corresponding to the different stimulus categories in the Mapper-generated shape graph.

For example, Figure 4 shows the shape graph generated and annotated for the first Haxby participant. The nodes corresponding to time frames during which the brain was processing images of faces (annotated using orange color) appear to be highly interconnected and localized in the shape graph. In comparison, the nodes corresponding to houses (annotated using green color) are localized in a different part of the graph and entirely disconnected from the orange-colored nodes for face stimuli. From this comparison, we might hypothesize that the brain processes these two categories quite differently. Supplemental Figure 3 (Supporting Information) provides shape graphs for all participants in the Haxby dataset.

The annotated shape graph can be further analyzed to study similarities within and between partitions of the data based on the connectivity of nodes in the shape graph. For example, Figure 4 (right panel) shows the similarity of data associated with visual stimuli within the same category and between different categories. This similarity matrix can be estimated for a single subject by examining connectivity of nodes within and between each stimulus category. For example, the heatmap shown in Figure 4 (right panel) implies slightly higher similarity of nodes associated with faces and cats stimuli, suggesting some overlap in how the brain processes and represents these stimuli categories in the ventral temporal cortex—reminiscent of the representational overlap proposed in the original study (Haxby et al., 2001). This example illustrates a potential use of the annotation property of DyNeuSR to both qualitatively and quantitatively examine hidden relationships between different event types in neuroimaging data.

Though annotation and visualization alone can provide some initial insight, it is important to interpret the shape graph in context of the data that were used to generate it. In this case, images of the brain can be mapped to the nodes for a more neuroscientific interpretation of brain processing across these categories, as discussed next.

#### Anchoring shape graphs into brain anatomy.

To anchor the shape graph properties into anatomy and neurophysiology, spatial maps of brain activity from samples (or time frames) within each shape graph node and the corresponding neighboring nodes can be averaged and overlaid onto an anatomical image of the brain. In addition to mere averaging, more advanced techniques like Spatial Mixture Modeling can also be used (Saggar et al., 2018).

**Figure 5.**
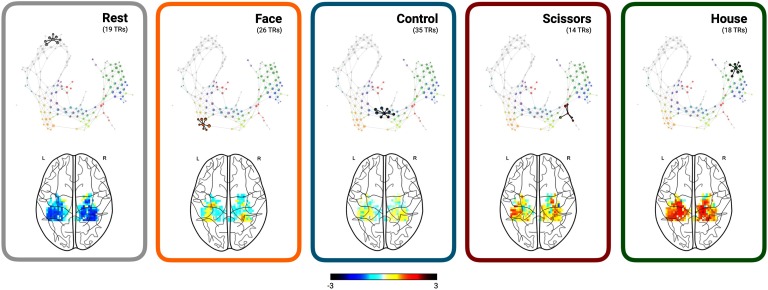
Anchoring the shape graph into brain anatomy. Here, average spatial maps of brain activity in the ventral temporal cortex estimated for individual time frames associated with different categories of visual stimuli are shown for the first subject from the Haxby study. Supplemental Figure 5 (Supporting Information) depicts this feature with the user interface.

Figure 5 shows several spatial brain activation patterns in the ventral stream estimated from nodes corresponding to resting state, three different categories of visual stimuli, and control stimuli (i.e., scrambled images). These anatomical overlays were estimated by averaging over spatial maps of brain activity associated with time frames sharing (or linking to) one or more similar nodes in the shape graph. It is important to note that because nodes in the shape graph contain samples (or time frames) that are very similar, and not necessarily sequential, averaging over these samples has been previously shown to be effective in capturing task-related brain activation patterns at the highest temporal resolution (Saggar et al., 2018).

Using the Haxby dataset as an example, one could explore how spatial maps of brain activity evolve over time as the participant transitions from one kind of stimuli to another. Further, the individual variations in such evolution could be used a potential biomarker for various mental disorders. In Supplemental Movie 1 (Supporting Information), we provide such an exploration of brain activation patterns over time extracted from the Mapper-generated shape graph.

#### Capturing temporal transitions in brain activity.

DyNeuSR can also be used to capture temporal dynamics and transitions in data based on the shape graph. To estimate temporal transitions in brain activity, the compressed shape graph can be transformed back to the original space of samples (or time frames). Such transformation then allows for examining relationships in the data at the highest level of temporal resolution (Saggar et al., 2018). This transformation is done by converting a Mapper-generated shape graph into an adjacency matrix in the temporal domain (i.e., a temporal connectivity matrix, TCM). Here, the time frames are considered connected (or similar) if they share a node in the shape graph or if the nodes containing these time frames are connected by an edge in the shape graph. Remarkably, the degree of TCM corresponds to the similarity of brain activation patterns captured at individual time frames and can be used to identify temporal transitions between different states of brain activity (at the level of individual time frames).

**Figure 6.**
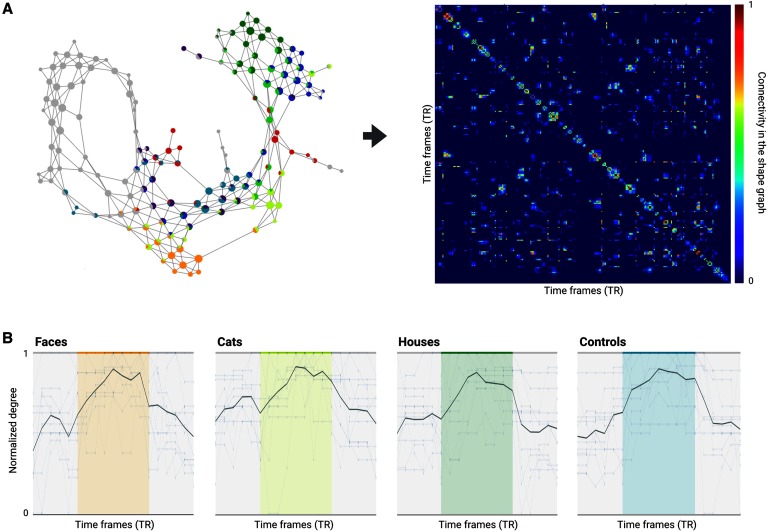
Capturing temporal transitions with shape graphs. (A) The shape graph and the temporal connectivity matrix (TCM) are shown for the first subject to illustrate how the shape graph can be projected back into the time domain. (B) The average temporal connectivity across subjects is shown for time frames associated with each of the different stimulus categories, illustrating how the degree of TCM can capture the onset and offset of stimulus blocks. The colored bars represent different categories of visual stimuli, and white space between colored bars represents rest.

Figure 6 shows the temporal connectivity matrix from the first Haxby participant. The onset, duration, and offset of stimulus blocks and the associated temporal connectivity and degree of TCM are shown for four different categories of visual stimuli. Qualitative analysis confirms that the temporal connectivity can reveal these temporal transitions at the level of individual time frames, and a time frame by time frame analysis suggests that the degree of TCM can capture both onset and offset of stimulus blocks for different categories of visual stimuli (colored bars in Figure 6). These results are similar to those reported by Saggar et al. (2018), where transitions between different types of cognitive tasks were captured from whole-brain activity. Our results help to validate this approach to characterizing dynamic aspects of brain activity. More work will be required, however, to extend the approach to capture transitions in brain activity when no stimulus information is provided, for example, during rest.

## DISCUSSION

In this paper, we introduce DyNeuSR, an open source Python platform for exploring, analyzing, and anchoring Mapper-generated topological graphs into known neurophysiological correlates. Specifically, DyNeuSR was designed to help users (a) annotate Mapper-generated shape graphs with meta-information; (b) anchor shape graphs to known neuroanatomical correlates; and (c) study topological properties of shape graphs to capture temporal transitions between coactivated brain patterns. We hope DyNeuSR will enable and encourage the larger neuroscience community to harness Mapper-based methods more effectively.

### Prior Work

Although TDA-based Mapper technique was recently developed (Singh et al., 2007), it has already been successfully applied to a variety of datasets. Outside of the neuroscience field, Mapper has been productively applied to reveal the shape of genetic data in breast cancer patients, where the shape graph was used to identify a unique subgroup of patients that exhibit 100% survival and no metastasis (Nicolau, Levine, & Carlsson, 2011). Mapper has also been used to characterize transient intermediate or transition states of biomolecular folding pathways (Yao et al., 2009), and to reveal implicit subgroups within the U.S. House of Representatives based on voting behavior (Lum et al., 2013).

Within neuroscience and neuroimaging fields, Mapper has been mostly employed at the population or group level to parse heterogeneity in data and identify subgroups that portray unique characteristics. For example, Mapper has been recently used to parse heterogeneity in patient populations and to identify clinically distinct neurophenotypes in children with fragile X syndrome (Bruno et al., 2017; Romano et al., 2014); patients with osteoarthritis (Pedoia et al., 2018; Rossi-deVries et al., 2018); patients with myalgic encephalomyelitis/chronic fatigue syndrome (Nagy-Szakal et al., 2017); inpatients with serious mental illness (Madan et al., 2017); patients with mild traumatic brain injury (Nielson et al., 2015); and individuals diagnosed with attention-deficit/hyperactivity disorder (Kyeong et al., 2015).

Unlike group-based studies, one unique feature of Mapper is that it allows users to extract insights at the level of individual participants, thereby helping users better understand individual differences in the brain’s dynamical organization (Saggar et al., 2018). Whereas standard neuroimaging analyses typically average or collapse the data into lower dimensional representations early on during the analysis, Mapper retains information about the original high-dimensional space throughout the analysis. Thus, the representations produced by Mapper could thereby provide novel measures of the neurophysiology underlying observed behavior. In the future, research should be done to assess whether Mapper-generated representations are robust and sensitive for predicting translational outcomes as well as for grounding psychiatric illnesses in biological mechanisms. DyNeuSR is designed to support both group-based and individual-based applications of Mapper.

Mapper has also been hypothesized to help visualize neural state-space traversal using dynamical networks (Khambhati, Sizemore, Betzel, & Bassett, 2018). As shown here, when applied to the Haxby visual decoding dataset, the DyNeuSR platform allows exploration of the continuous unfolding of brain dynamics across each time frame. Further, by allowing annotation of shape graphs based on meta-information (e.g., category of visual stimuli presented), DyNeuSR also enables multimodal data exploration. Lastly, individual nodes in the shape graph can be anchored into neurophysiological correlates and examined across the period of one or more scans using interactive tools provided in the DyNeuSR platform.

### Future Applications

Over the last 10 years, there has been a huge surge in collecting and aggregating spatiotemporally rich neuroimaging data. Further, advances in technology have enabled more sharing of data across the world. In 2014, more than 8,000 MRI datasets alone were shared online (Poldrack & Gorgolewski, 2014). To take advantage of this widespread access to spatiotemporally rich high-dimensional datasets, novel methods are needed to extract computational insights that are robust against noise, reproducible, and biologically valid. The ultimate aim is to produce methods that can translate neuroscientific knowledge into clinical applications.

We argue that methods like TDA-based Mapper are well-suited for this challenge. As shown previously, Mapper can be employed at the population level to parse out heterogeneity and reveal the underlying structure of the data when applied at the individual level. Further, unlike traditional machine learning algorithms, Mapper makes fewer assumptions about the underlying data; represents the underlying structure as a combinatorial object (i.e., graph), whose topological properties can be easily quantified; and provides coordinate and deformation invariance properties, making it suitable for examining within and between participants’ data (Carlsson, 2009). Lastly, because of the partial clustering step, Mapper-based analysis is computationally robust to handle very large datasets.

Cognitive and clinical neuroscientists have started incorporating macroscopic imaging methods into assessments of brain dynamics to develop more personalized diagnostic and therapeutic strategies (Williams, 2017). DyNeuSR anchors topological data analysis into anatomical structure, enabling potentially quick data analysis for practical clinical use. For example, there are many cases of brain dysfunction or disease that manifest as changes in the dynamical landscape of brain activity (Du et al., 2018, 2017). Thus, it should be possible to discriminate between these dysfunctional brain dynamics and disordered cognitive states, and DyNeuSR has the potential to enable such discrimination in a research or clinical setting.

Topological data analysis offers compact and insightful visualization of high-dimensional neuroimaging data, and as a result, facilitates the characterization of large-scale structural and functional brain networks. Although the examples presented here focus on fMRI data, the core components of DyNeuSR can be easily extended to a diversity of other applications and data types. For example, future research plans include extending DyNeuSR to structural connectivity estimated from diffusion tensor imaging (DTI). It should also be noted that TDA approaches are amenable to other sources of neuroscience data as well, including invasive neurophysiological, optical imaging, and spike recordings obtained from model organisms.

### Limitations

One of the major limitations of Mapper is that the topology of shape graph highly depends on the chosen filter function (e.g., linear vs. nonlinear) and resolution/gain parameters. While the topological observables are typically robust by construction to parameter perturbations, as shown in Saggar et al. (2018), it would be still very useful to quantify the effect of perturbations in parameter or filter space. Carrière, Michel, & Oudot (2018) attempted to automate parameter selection; however, it remains unclear how to compare Mapper graphs obtained from different filter functions. Such comparisons would likely require information-theoretic methods based on clustering comparison (e.g., Gates, Wood, Hetrick, & Ahn, 2017), or graph-size agnostic topological methods, such as distances in persistent homology (Bergomi, Ferri, & Zuffi, 2017; Reininghaus, Huber, Bauer, & Kwitt, 2015). Further, random null models that constrain specific properties of the Mapper graph can be used to establish the significance of the observed features, analogous to how random network models are used in network science. While random topological models have been proposed (Costa & Farber, 2016; Kahle, 2009; Zuev, Eisenberg, & Krioukov, 2015), few can be directly compared with the (typically irregular) structures obtained from data (e.g., Young, Petri, Vaccarino, & Patania, 2017). Consequently, the inability to make these direct comparisons could potentially impede statistical validation of Mapper results.

Nonetheless, like any other machine learning technique, interpretation of Mapper results should always be taken in the context of model parameters. Further, choice of filter functions and other Mapper parameters also make this tool extremely versatile for both data exploration and feature extraction in large, complex datasets (Phinyomark et al., 2017), for example by providing topological maps of feature spaces that can guide the choice of relevant features. Ultimately, the choice of filter function depends on both the data itself and the research questions at hand. For example, Supplemental Figure 2 (Supporting Information) shows how using PCA as a linear filter function, Mapper is able to capture global differences in the data, such as rest versus stimuli presentation. In addition to this global structure in the data, however, using t-SNE as a nonlinear filter function, Mapper is able to capture additional local structure in the data, as indicated by more fine-scale differentiation of different stimulus categories.

### Conclusions

DyNeuSR is still in the early stages of development, and it has the potential to grow in exciting and novel ways. Some future research directions include (a) developing a new filter function for neuroimaging data that utilizes structural connectome; (b) linking the neurophysiological correlates obtained from the Mapper shape graph to standard dynamic functional connectivity; and (c) applying persistent homology to analyze DyNeuSR results.

As we continue to develop and refine DyNeuSR, we invite others to contribute. By making DyNeuSR an open source tool, we hope that future work on DyNeuSR will be a collaborative and productive effort. Thus, to accelerate such efforts and encourage distributed open source development, our priorities include refactoring, testing, and documenting the codebase. Future code development will also include integrating other tools from topological data analysis.

To help share this software with the neuroimaging community, we plan on developing additional tutorials and examples demonstrating the use of Mapper in other neuroimaging datasets. For example, in other disciplines, researchers often leverage Mapper to visualize high-dimensional datasets and detect outliers in the data. We plan on exploring how researchers can leverage DyNeuSR’s visualization tools to detect outliers in raw fMRI data during preprocessing. We hope researchers can use DyNeuSR in combination with other software packages for fMRI data preprocessing, such as fMRIPrep (Esteban et al., 2018) and C-PAC (Sikka et al., 2014).

We think DyNeuSR can offer unique insight into neuroimaging data analysis and visualization. To this end, we also hope to integrate DyNeuSR with widely used Python software, such as Nilearn (nilearn.github.io) and nipy (github.com/nipy/nipy). Finally, to improve the descriptive power of DyNeuSR, we welcome the addition of network and simplicial packages within DyNeuSR’s analytical framework, such as graph-tool (graph-tool.skewed.de), GUDHI (gudhi.gforge.inria.fr), and SNAP (snap.stanford.edu).

## MATERIALS AND METHODS

### Datasets

We used the following two datasets: (a) synthetic 3-D trefoil knot data; and (b) real neuroimaging data from the Haxby visual decoding experiment. Below, we will briefly describe each of these datasets.

#### Trefoil knot.

The first dataset consists of a synthetic set of data points sampled from a three-dimensional trefoil knot. The space is parameterized by a set of *sin* and *cos* functions over the domain (0, 2*π*) defining the position of *n* sampled points in *x*−, *y*−, and *z*−dimensions. Specifically, the following equations describe the parameters:

$$\begin{array}{c} \phi \in [0,2\pi ]\\ x=\sin\left(\phi \right)+2\cos\left(\text{2}\phi \right)\\ y=\sin(\phi )-2\cos\left(2\phi \right)\\ z=-\sin\left(\text{3}\phi \right) \end{array}$$

The code to sample data from these equations was implemented using the Numerical Python (NumPy) software package. To see the Python code used to load and prepare the data, please refer to the Jupyter notebook provided in Supplemental Data 1 (Supporting Information).

#### Haxby fMRI dataset.

We also explored a fMRI dataset from a study on face and object recognition in the human ventral temporal cortex (Haxby et al., 2001). In this study, visual stimuli from eight different categories were shown to six different subjects over 12 sessions per subject. In each session, fMRI data were collected while subjects passively viewed grayscale images from the eight different categories grouped into stimulus blocks and separated by periods of rest. Specifically, during each 24-s block of images, each image was presented for 500 ms and followed by an interstimulus interval (ISI) of 1,500 ms. The data were collected with a volume repetition time (TR) of 2,500 ms, which means around nine volumes were collected for each stimulus category per session. The fMRI data were stored as a 4-D NIfTI time series image, consisting of 1,452 volumes with 40 × 64 × 64 voxels (i.e., corresponding to a voxel size = 3.5 × 3.75 × 3.75 mm). A NIfTI image mask of the ventral temporal cortex was used to extract a subset of voxels relevant to visual decoding.

To download the Haxby dataset, we used Nilearn’s API. We also used Nilearn to load and preprocess the NIfTI time series images and extract NumPy matrix representations for input to Mapper. For each of the six subjects, we used as the input to Mapper a matrix with time frames as rows and voxels as columns. Specifically, we used 242 time frames from Sessions 4 and 5, and we used voxels from each subjects’ ventral temporal cortex (e.g., 577 voxels for the first subject, 348 voxels for the sixth subject). We used subject-specific NIfTI anatomical masks provided with the dataset to identify the specific voxels corresponding to each subjects’ ventral temporal cortex. We used Stochastic Neighborhood Embedding (t-SNE; Maaten & Hinton, 2008) as a lens for filtering, and a resolution of 17 and gain of 3 (i.e., 66.7% overlap) as cover parameters for binning. To see the Python code used to load and prepare the data, please refer to the Jupyter notebook provided in Supplemental Data 2 (Supporting Information).

### Mapper Implementation

In this work, we generated all shape graphs with the open source KeplerMapper Python package (Saul & van Veen, 2017).

### Overview of DyNeuSR

DyNeuSR is a Python package for interactive graph visualization and neuroscientific analysis of Mapper-generated shape graphs.

#### Installation.

DyNeuSR can be installed as a Python module and imported into any Python environment. The current implementation requires Python 3.

#### Visualizing and interacting with shape graphs.

The primary way to visualize and interact with the shape graph is through DyNeuSR’s web-based interface. This interface is automatically generated using a combination of HTML, JavaScript, and D3. As shown in Supplemental Figure 4 (Supporting Information), the interface is composed of three main parts: (a) an annotated shape graph with a force-directed layout; (b) a panel with information about the shape graph (e.g., source file name, color legend); and (c) a hover box that displays information about individual nodes (e.g., labels). Moreover, every node assigned more than a single color (or annotation) is represented as a pie chart with slices proportional to the number of samples in each group.

#### Anchoring shape graphs into brain anatomy.

To anchor the Mapper-generated graphical representation in neurophysiology, DyNeuSR provides different ways to estimate and visualize representative brain activation (and deactivation) maps for each node in the shape graph. Users can generate plots of the estimated brain activation patterns using Nilearn, and the resulting images can be included in the hover box assigned to each node in the shape graph.

#### Capturing temporal transitions in brain activity.

DyNeuSR provides methods for capturing temporal dynamics and transitions in data based on the shape graph. By default, after constructing a NetworkX graphical object representation of the shape graph, DyNeuSR creates a temporal connectivity matrix (TCM) based on the node membership and connectivity of time frames (or samples). Specifically, the TCM encodes the similarity between all the time frames (or across samples). The degree of nodes in the TCM can then be used to identify temporal transitions associated with task-related brain activity (Saggar et al., 2018). For each node in the TCM, the degree is estimated as the number of connecting nonzero edges. This approach can also be used to describe the temporal (or sample) evolution of other graph metrics. At this time, DyNeuSR only offers tools for estimating temporal connectivity using the normalized degree of nodes in the shape graph. However, the code could easily be extended to leverage other graph measures.

## SUPPORTING INFORMATION

DyNeuSR is an open source project. The code, documentation, and example tutorials are publicly available at http://bdl.stanford.edu/projects/dyneusr.

Supplementary figures, data, and movies are available at https://doi.org/10.1162/netn_a_00093.

## AUTHOR CONTRIBUTIONS

Caleb Geniesse: Investigation; Methodology; Software; Visualization; Writing - Review & Editing. Olaf Sporns: Methodology; Validation; Writing - Review & Editing. Giovanni Petri: Methodology; Validation; Writing - Review & Editing. Manish Saggar: Conceptualization; Data curation; Funding acquisition; Investigation; Methodology; Project administration; Software; Supervision; Validation; Visualization; Writing - Original Draft; Writing - Review & Editing.

## FUNDING INFORMATION

Manish Saggar, National Institute of Mental Health (http://dx.doi.org/10.13039/100000025), Award ID: R00 MH104605. Caleb Geniesse, National Institute of General Medical Sciences (http://dx.doi.org/10.13039/100000057), Award ID: T32 GM008294. Manish Saggar, National Institutes of Health (http://dx.doi.org/10.13039/100000002), Award ID: DP2 MH119735.

## TECHNICAL TERMS

Topological data analysis (TDA): Applied mathematical approach to analyzing datasets using techniques from algebraic topology and providing information about the shape of complex data.
Mapper: TDA-based technique for extracting graphical descriptions of point cloud data based on the idea of filter-guided partial clustering.
Shape graph: Compressed network representation generated by Mapper; nodes correspond to local clusters and edges connect nodes that share data points.
Dimensionality reduction: Process of selecting and extracting features from data to reduce the number of random variables under consideration.
Cover: Set of overlapping bins of data points produced by the partitioning of the data along one or more filters defined on that data.
Filtering: First stage of Mapper where low-dimensional views (embeddings) of data are constructed in order to guide partial clustering in later stages.
Stochastic Neighborhood Embedding: Nonlinear method for projecting high-dimensional data into a low-dimensional space in a way that preserves local structure in the original space.

## References

[SMS5] DyNeuSR DyNeuSR http://bdl.stanford.edu/projects/dyneusr (2018).

[bib1] Bergomi M. G., Ferri M., & Zuffi L. (2017). Topological graph persistence. arXiv:1707.09670.

[bib2] Bruno J. L., Romano D., Mazaika P., Lightbody A. A., Hazlett H. C., Piven J., & Reiss A. L. (2017). Longitudinal identification of clinically distinct neurophenotypes in young children with fragile X syndrome. Proceedings of the National Academy of Sciences, 114(40), 10767–10772. 10.1073/pnas.1620994114 PMC5635864 28923933

[bib3] Carlsson G. (2009). Topology and data. Bulletin of the American Mathematical Society, 46(2), 255–308.

[bib4] Carrière M., Mitchel B., & Oudot S. (2018). Statistical analysis and parameter selection for mapper. Journal of Machine Learning Research, 19, 1–39.

[bib5] Costa A., & Farber M. (2016). Random simplicial complexes. In Callegaro F., Cohen F., De Concini C., Feichtner E., Gaiffi G., & Salvetti M. (Eds.), Configuration spaces (Springer INdAM series, Vol. 14, pp. 129–153). Cham, Switzerland: Springer International Publishing

[bib6] Du Y., Fryer S. L., Fu Z., Lin D., Sui J., Chen J., … Calhoun V. D. (2018). Dynamic functional connectivity impairments in early schizophrenia and clinical high-risk for psychosis. NeuroImage, 180, 632–645. 29038030 10.1016/j.neuroimage.2017.10.022 PMC5899692

[bib7] Du Y., Pearlson G. D., Lin D., Sui J., Chen J., Salman M., … Calhoun V. D. (2017). Identifying dynamic functional connectivity biomarkers using GIG-ICA: Application to schizophrenia, schizoaffective disorder, and psychotic bipolar disorder. Human Brain Mapping, 38(5), 2683–2708. 28294459 10.1002/hbm.23553 PMC5399898

[bib8] Esteban O., Markiewicz C., Blair R. W., Moodie C., Isik A. I., Aliaga A. E., … Gorgolewski K. J. (2018). FMRIPrep: A robust preprocessing pipeline for functional MRI. bioRxiv:306951. 10.1038/s41592-018-0235-4 PMC6319393 30532080

[bib9] Gates A. J., Wood I. B., Hetrick W. P., & Ahn Y.-Y. (2017). On comparing clusterings: An element-centric framework unifies overlaps and hierarchy. arXiv:1706.06136. 10.1038/s41598-019-44892-y PMC6561975 31189888

[bib10] Geniesse C., Sporns O., Petri G., & Saggar M. (2018). Generating dynamical neuroimaging spatiotemporal representations (DyNeuSR) using topological data analysis. http://bdl.stanford.edu/projects/dyneusr 10.1162/netn_a_00093 PMC6663215 31410378

[bib11] Haxby J. V., Gobbini M. I., Furey M. L., Ishai A., Schouten J. L., & Pietrini P. (2001). Distributed and overlapping representations of faces and objects in ventral temporal cortex. Science, 293(5539), 2425–2430. 11577229 10.1126/science.1063736

[bib12] Kahle M. (2009). Topology of random clique complexes. Discrete Mathematics, 309(6), 1658–1671.

[bib13] Karahanoğlu F. I., & Van De Ville D. (2015). Transient brain activity disentangles fMRI resting-state dynamics in terms of spatially and temporally overlapping networks. Nature Communications, 6(1), 7751 10.1038/ncomms8751 PMC4518303 26178017

[bib14] Khambhati A. N., Sizemore A. E., Betzel R. F., & Bassett D. S. (2018). Modeling and interpreting mesoscale network dynamics. NeuroImage, 180(Pt. B), 337–349. 28645844 10.1016/j.neuroimage.2017.06.029 PMC5738302

[bib15] Kyeong S., Park S., Cheon K.-A., Kim J.-J., Song D.-H., & Kim E. (2015). A new approach to investigate the association between brain functional connectivity and disease characteristics of attention-deficit/hyperactivity disorder: Topological neuroimaging data analysis. PloS ONE, 10(9), e0137296 26352147 10.1371/journal.pone.0137296 PMC4564101

[bib16] Liu X., & Duyn J. H. (2013). Time-varying functional network information extracted from brief instances of spontaneous brain activity. Proceedings of the National Academy of Sciences, 110(11), 4392–4397. 10.1073/pnas.1216856110 PMC3600481 23440216

[bib17] Liu X., Zhang N., Chang C., & Duyn J. H. (2018). Co-activation patterns in resting-state fMRI signals. NeuroImage, 180(Pt. B), 485–494. 29355767 10.1016/j.neuroimage.2018.01.041 PMC6082734

[bib18] Lum P. Y., Singh G., Lehman A., Ishkanov T., Vejdemo-Johansson M., Alagappan M., … Carlsson G. (2013). Extracting insights from the shape of complex data using topology. Scientific Reports, 3, 1236 23393618 10.1038/srep01236 PMC3566620

[bib19] Maaten L., & Hinton G. (2008). Visualizing data using t-SNE. Journal of Machine Learning Research, 9, 2579–2605.

[bib20] Madan A., Fowler J. C., Patriquin M. A., Salas R., Baldwin P. R., Velasquez K. M., … Frueh C. (2017). A novel approach to identifying a neuroimaging biomarker for patients with serious mental illness. Journal of Neuropsychiatry and Clinical Neurosciences, 29(3), 275–283. 28238273 10.1176/appi.neuropsych.16090174

[bib21] Nagy-Szakal D., Williams B. L., Mishra N., Che X., Lee B., Bateman L., … Lipkin W. I. (2017). Fecal metagenomic profiles in subgroups of patients with myalgic encephalomyelitis/chronic fatigue syndrome. Microbiome, 5(1), 44 28441964 10.1186/s40168-017-0261-y PMC5405467

[bib22] Nicolau M., Levine A. J., & Carlsson G. (2011). Topology based data analysis identifies a subgroup of breast cancers with a unique mutational profile and excellent survival. Proceedings of the National Academy of Sciences, 108(17), 7265–7270. 10.1073/pnas.1102826108 PMC3084136 21482760

[bib23] Nielson J. L., Paquette J., Liu A. W., Guandique C. F., Tovar C. A., Inoue T., … Ferguson A. R. (2015). Topological data analysis for discovery in preclinical spinal cord injury and traumatic brain injury. Nature Communications, 6, 8581 10.1038/ncomms9581 PMC4634208 26466022

[bib24] Pedoia V., Haefeli J., Morioka K., Teng H.-L., Nardo L., Souza R. B., … Majumdar S. (2018). MRI and biomechanics multidimensional data analysis reveals R_2_-R_1_ρ as an early predictor of cartilage lesion progression in knee osteoarthritis. Journal of Magnetic Resonance Imaging, 47(1), 78–90. 28471543 10.1002/jmri.25750 PMC5967390

[bib25] Phinyomark A., Ibanez-Marcelo E., & Petri G. (2017). Resting-state fMRI functional connectivity: Big data preprocessing pipelines and topological data analysis. IEEE Transactions on Big Data, 3(4), 415–428.

[bib26] Poldrack R. A., & Gorgolewski K. J. (2014). Making big data open: Data sharing in neuroimaging. Nature Neuroscience, 17(11), 1510–1517. 25349916 10.1038/nn.3818

[bib27] Preti M. G., Bolton T. A., & Van De Ville D. (2016). The dynamic functional connectome: State-of-the-art and perspectives. NeuroImage. 10.1016/j.neuroimage.2016.12.061 28034766

[bib28] Reininghaus J., Huber S., Bauer U., & Kwitt R. (2015). A stable multi-scale kernel for topological machine learning (Vol. 7, pp. 4741–4748). Presented at the Proceedings of the IEEE Computer Society Conference on Computer Vision and Pattern Recognition, IEEE

[bib29] Romano D., Nicolau M., Quintin E.-M., Mazaika P. K., Lightbody A. A., Cody Hazlett H., … Reiss A. L. (2014). Topological methods reveal high and low functioning neuro-phenotypes within fragile X syndrome. Human Brain Mapping. 10.1002/hbm.22521 PMC4113391 24737721

[bib30] Rossi-deVries J., Pedoia V., Samaan M. A., Ferguson A. R., Souza R. B., & Majumdar S. (2018). Using multidimensional topological data analysis to identify traits of hip osteoarthritis. Journal of Magnetic Resonance Imaging, 48(4), 1046–1058. 29734501 10.1002/jmri.26029 PMC6174097

[bib31] Saggar M., Sporns O., Gonzalez-Castillo J., Bandettini P. A., Carlsson G., Glover G., & Reiss A. L. (2018). Towards a new approach to reveal dynamical organization of the brain using topological data analysis. Nature Communications, 9(1), 1399. 10.1038/s41467-018-03664-4 PMC5895632 29643350

[bib32] Saul N., & van Veen H. J. (2017). MLWave/kepler-mapper: 186f. Zenodo.

[bib33] Shine J. M., Koyejo O., Bell P. T., Gorgolewski K. J., Gilat M., & Poldrack R. A. (2015). Estimation of dynamic functional connectivity using Multiplication of Temporal Derivatives. NeuroImage, 122, 399–407. 26231247 10.1016/j.neuroimage.2015.07.064

[bib34] Sikka S., Cheung B., Khanuja R., Ghosh S., Yan C., Li Q., … Milham M. (2014). Towards automated analysis of connectomes: The configurable pipeline for the analysis of connectomes (C-PAC). Frontiers in Neuroinformatics. Conference abstract: 5th INCF Congress of Neuroinformatics

[bib35] Singh G., Mémoli F., & Carlsson G. E. (2007). Topological methods for the analysis of high dimensional data sets and 3d object recognition. Symposium on Point Based Graphics.

[bib36] Williams L. M. (2017). Getting personalized: Brain scan biomarkers for guiding depression interventions. American Journal of Psychiatry, 174(6), 503–505. 28565957 10.1176/appi.ajp.2017.17030314

[bib37] Xu Y., & Lindquist M. A. (2015). Dynamic connectivity detection: An algorithm for determining functional connectivity change points in fMRI data. Frontiers in Neuroscience, 9(143), 285 26388711 10.3389/fnins.2015.00285 PMC4560110

[bib38] Yao Y., Sun J., Huang X., Bowman G. R., Singh G., Lesnick M., … Carlsson G. (2009). Topological methods for exploring low-density states in biomolecular folding pathways. Journal of Chemical Physics, 130(14), 144115 19368437 10.1063/1.3103496 PMC2719471

[bib39] Young J. G., Petri G., Vaccarino F., & Patania A. (2017). Construction of and efficient sampling from the simplicial configuration model. Physical Review E, 96(3), 477 10.1103/PhysRevE.96.032312 29346916

[bib40] Zuev K., Eisenberg O., & Krioukov D. (2015). Exponential random simplicial complexes. Journal of Physics A: Mathematical and Theoretical, 48(46), 465002

