# Supplementary material for: Generating dynamical neuroimaging spatiotemporal representations (DyNeuSR) using topological data analysis
Source: Netw Neurosci. 2019 Jul 1;3(3):763–78. doi: 10.1162/netn_a_00093 (PMC6663215; doi:10.1162/netn_a_00093)
Supplement: Supplementary file 1 [file netn-03-763-s001.pdf]

Supplement to: Generating dynamical neuroimaging spatiotemporal representations (DyNeuSR) using topological data analysis

Caleb Geniesse[1,2], Olaf Sporns[3], Giovanni Petri[4,5], Manish Saggar[1,2*]

[1]Biophysics Program, Stanford University

[2]Department of Psychiatry & Behavioral Sciences, Stanford University

[3]Department of Psychological and Brain Sciences, Indiana University

[4]ISI Foundation, Turin, Italy

[5]ISI Global Science Foundation, New York, USA

Keywords: brain dynamics, TDA, fMRI, brain networks, Mapper

*Corresponding Author

Supplemental Figures

Here we provide figures as supplemental information for the main text. Supplemental figures include:

1. Comparison of Mapper parameters on the trefoil knot.
2. Comparison of Mapper filter functions on Haxby dataset.
3. Comparison of shape graphs across subjects from the Haxby dataset.
4. Preview of DyNeuSR's interactive interface for shape graph visualization.
5. Preview of DyNeuSR's interface after anchoring the shape graph to brain anatomy.

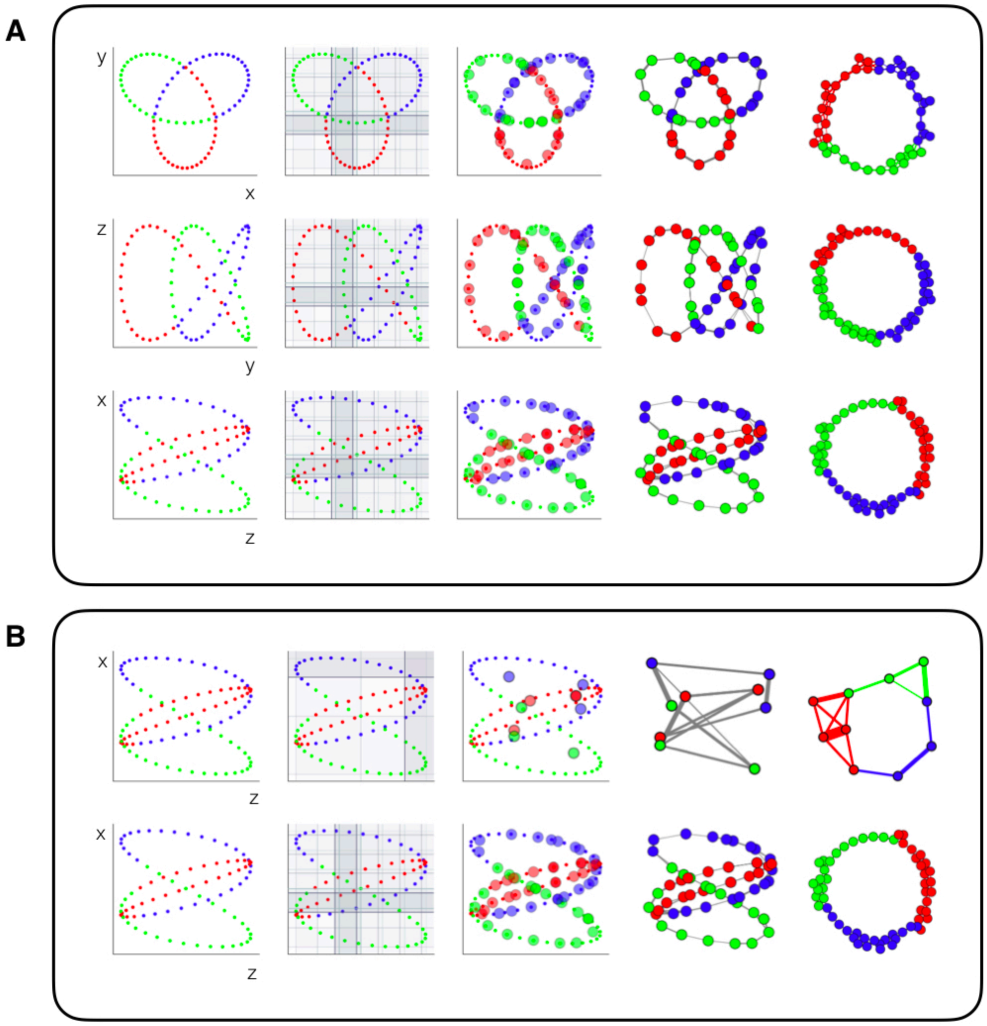

Supplemental Figure 1. *Comparison of Mapper parameters on the trefoil knot.* Mapper robustly captures the intrinsic shape of data. The trefoil knot is a one-dimensional manifold (i.e., its intrinsic shape is a circle) and Mapper recovers similar representations of this intrinsic circular shape (even when using different lenses on the data and/or different resolution coverings). (**A**) The intermediate stages and the final shape graphs generated by Mapper using different pairs of columns as a lens (i.e., two-dimensional projections of the three-dimensional data). (**B**) Similarly, the results generated by Mapper using low- and high-resolution coverings (i.e., number of bins).

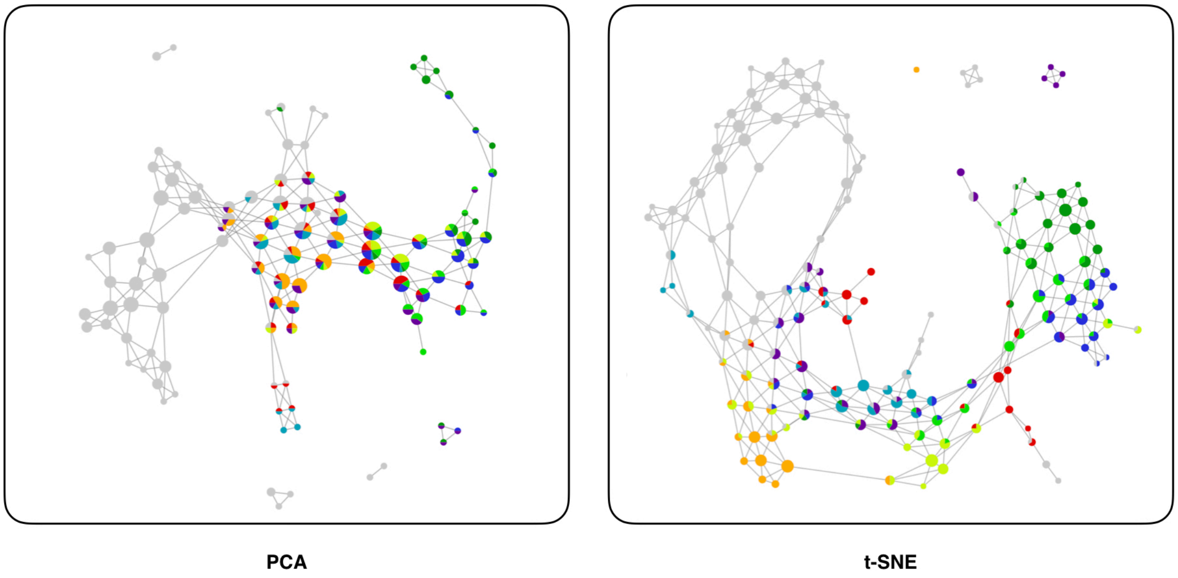

Supplemental Figure 2. *Comparison of Mapper filter functions on the Haxby dataset.* The shape graphs generated by Mapper using linear (e.g., PCA) and non-linear (e.g., t-SNE) filter functions are shown for the first subject from the Haxby dataset. Using PCA as the filter function, Mapper is able to capture global differences in the data, such as Rest (gray) vs. visual stimulus (color). In addition to this global structure in the data, however, using t-SNE as the filter function, Mapper is able to capture additional local structure in the data, as indicated by more fine-scale differentiation of different categories as visual stimuli (i.e., more homogenous coloring of the nodes corresponds to better separation of the different categories).

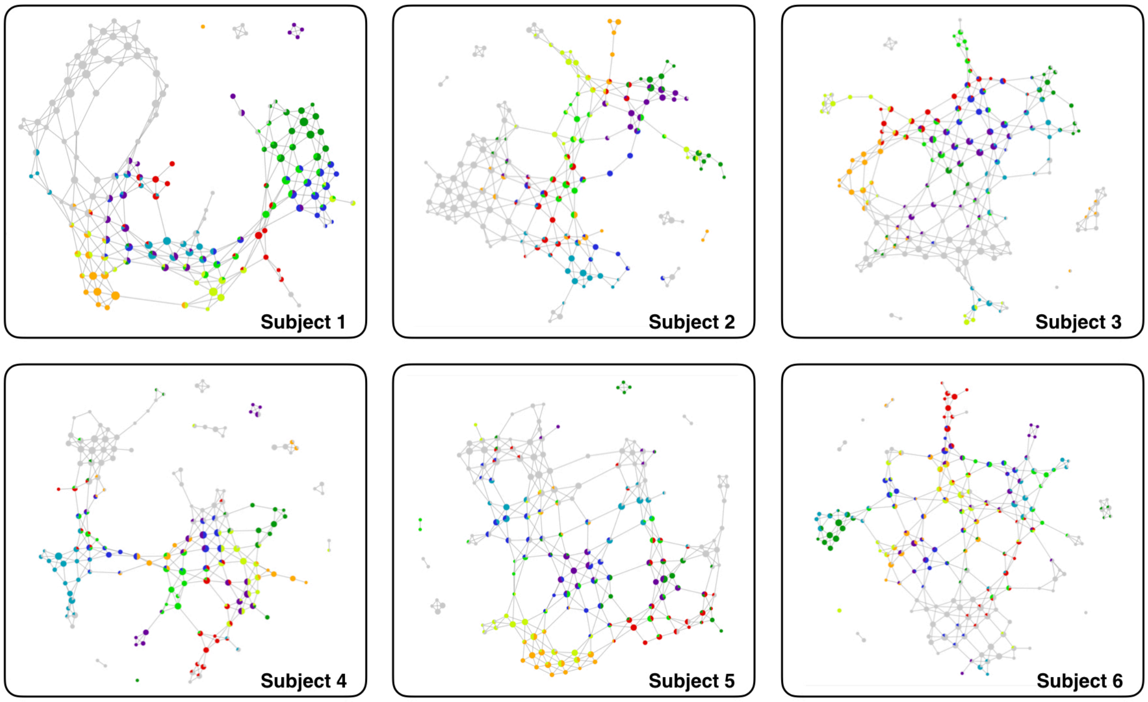

Supplemental Figure 3. *Comparison of shape graphs across subjects from the Haxby dataset.* The shape graphs generated by Mapper using a non-linear (e.g., t-SNE) filter function are shown for all 6 subjects (sessions 4 and 5) from the Haxby dataset.

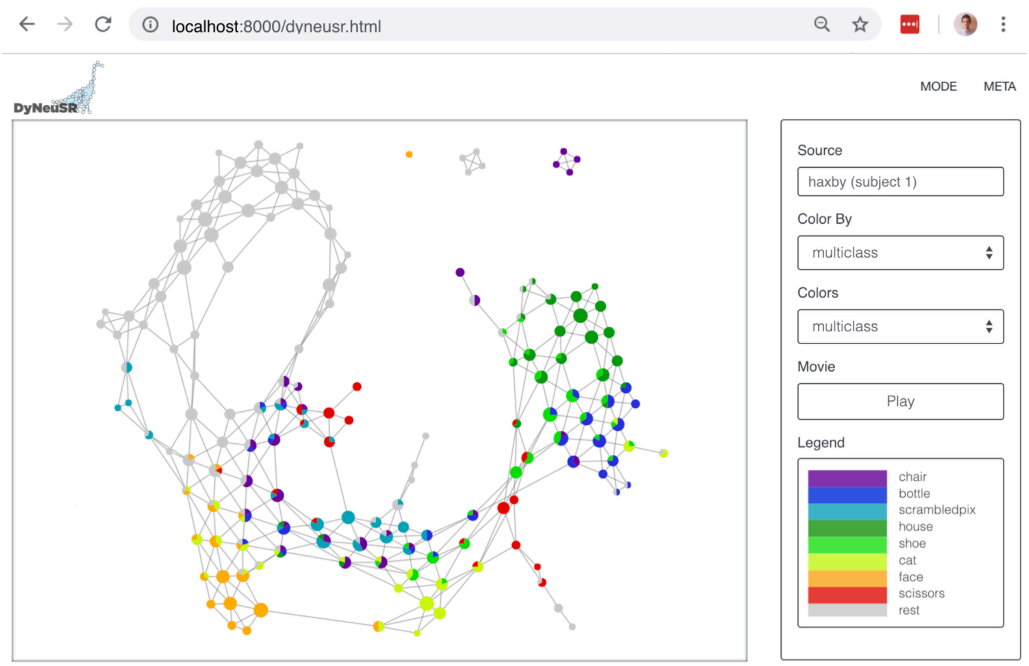

Supplemental Figure 4. *Preview of DyNeuSR's interactive interface for shape graph visualization.* DyNeuSR provides a user interface based on D3.js for interactive visualization of shape graphs. Through this interface, a user can toggle different aspects of (and interact with) the data by hovering over or dragging the shape graph. A user can select a single node to display (in the lower right corner) meta-information and annotations corresponding to the data points associated with the selected node.

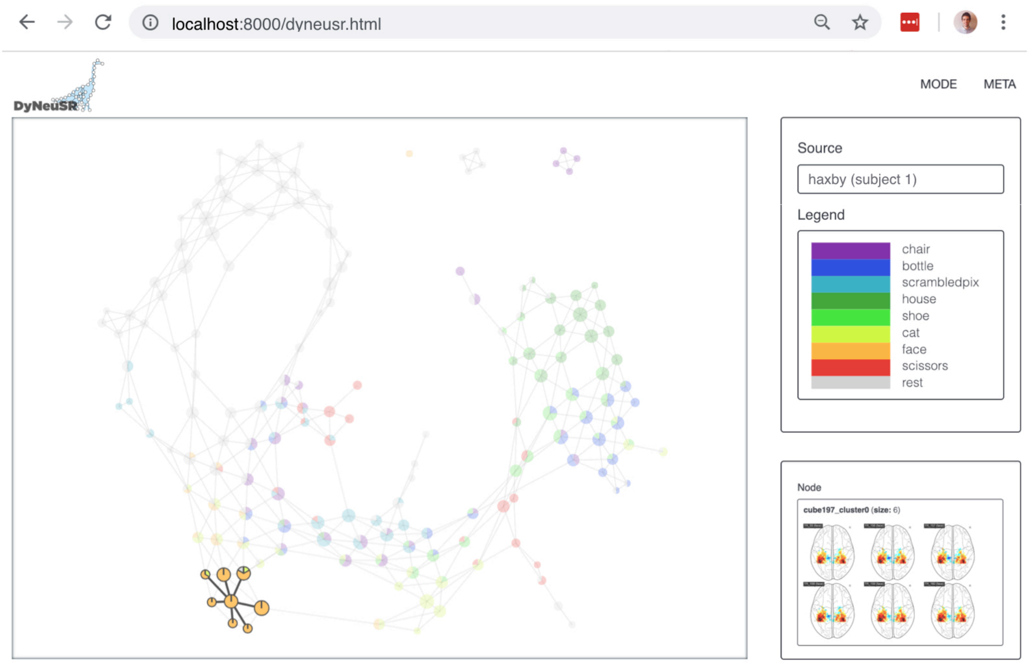

Supplemental Figure 5. *Preview of DyNeuSR's interface after anchoring the shape graph to brain anatomy.* DyNeuSR's interface also allows for interactive visualization of the shape graph after it has been anchored to brain anatomy. To anchor the shape graph properties into neurophysiology, a user can select a single node to display (in the lower right corner) spatial maps of brain activity overlaid onto brain anatomy for different time frames associated with the node.

Supplemental Movie 1. *Movie of brain images generated with DyNeuSR.*
