# Supplementary material for: Generating dynamical neuroimaging spatiotemporal representations (DyNeuSR) using topological data analysis
Source: Netw Neurosci. 2019 Jul 1;3(3):763–78. doi: 10.1162/netn_a_00093 (PMC6663215; doi:10.1162/netn_a_00093)
Supplement: Supplementary file 2 [file netn-03-763-suppData_1.pdf]

Geniesse, C., Sporns, O., Petri, G., & Saggar, M. (2019). Supporting information for "Generating dynamical neuroimaging spatiotemporal representations (DyNeuSR) using topological data analysis." *Network Neuroscience*, 3(3), 763–778. https://doi.org/10.1162/netn_a_00093

# 01_trefoil_knot

February 25, 2019

## 1 Example 1: Visualizing a trefoil knot with `kmapper` + `dyneusr`

### 1.1 1 `import` libraries

In [1]:
```
# for auto-reloading external modules
%load_ext autoreload
%autoreload 2
```

In [2]:
```
import numpy as np
import pandas as pd
import scipy as sp

import networkx as nx
from collections import Counter
```

```
/Users/saggar/anaconda3/lib/python3.6/site-packages/h5py/__init__.py:36: FutureWarning: Convers
  from ._conv import register_converters as _register_converters
```

In [3]:
```
import matplotlib as mpl
import matplotlib.pyplot as plt
import seaborn as sns
sns.set("paper", "white")

%matplotlib inline
```

#### 1.1.1 1.1 `import kmapper`

Here, we will use the KeplerMapper (`kmapper`) implementation of the Mapper algorithm.

We will also import sklearn implementations of PCA and TSNE to see how these standard (linear and non-linear, respectively) dimensionality reduction tools compare to Mapper.

In [4]:
```
import kmapper as km
from sklearn.decomposition import PCA
from sklearn.manifold import TSNE
```

### 1.1.2 1.2 import dyneusr

Note, all code to generate and visualize a synthetic trefoil knot, used below, is provided by the dyneusr.datasets module.

```
In [5]: import dyneusr as ds

        # trefoil datasets
        from dyneusr.datasets.trefoil import make_trefoil, Bunch
        from dyneusr.datasets.trefoil import draw_trefoil3d, draw_trefoil

        # visualizing the stages of mapper
        from dyneusr.tools.networkx_utils import visualize_mapper_stages
```

## 1.2 2 Load data

```
In [6]: # sample 100 points from the trefoil knot
        data = make_trefoil(size=100)

        # print some info
        print("Dataset (keys):", data.keys())
        print("Data has shape:", data.data.shape)
```

```
Dataset (keys): dict_keys(['data', 'feature_names', 'target', 'coloring', 'cmap', 'norm', 'inde
Data has shape: (100, 3)
```

```
In [7]: # define inputs: X=data, y=meta
        X = data.data
        X_inverse = data.data
        y = data.target
        c = data.coloring

        # visualize each dimension of the trefoil knot
        fig, axes = plt.subplots(1, 1, figsize=(15, 2))
        for i,x in enumerate(X.T):
            plt.scatter(np.arange(x.size), x, c=c)
```

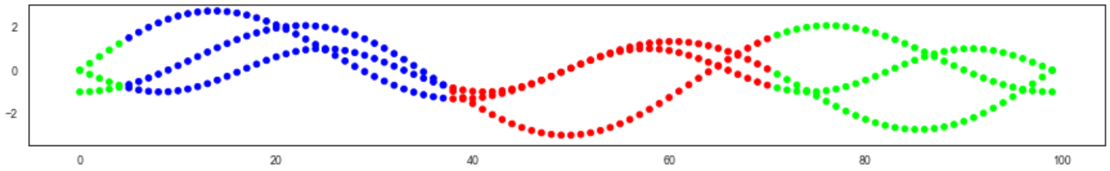

### 1.2.1 2.1 Visualize the data

```
In [8]: # visualize each dimension of the trefoil knot
        _ = draw_trefoil(*data.data.T, c=data.coloring)

        # visualize 3D scatter plots of the trefoil knot
        _ = draw_trefoil3d(*data.data.T, c=data.coloring)
        _ = draw_trefoil3d(*data.data.T, c=data.coloring, view=(60, -90))
        _ = draw_trefoil3d(*data.data.T, c=data.coloring, view=(30, -90))
        _ = draw_trefoil3d(*data.data.T, c=data.coloring, view=(0, -90))
```

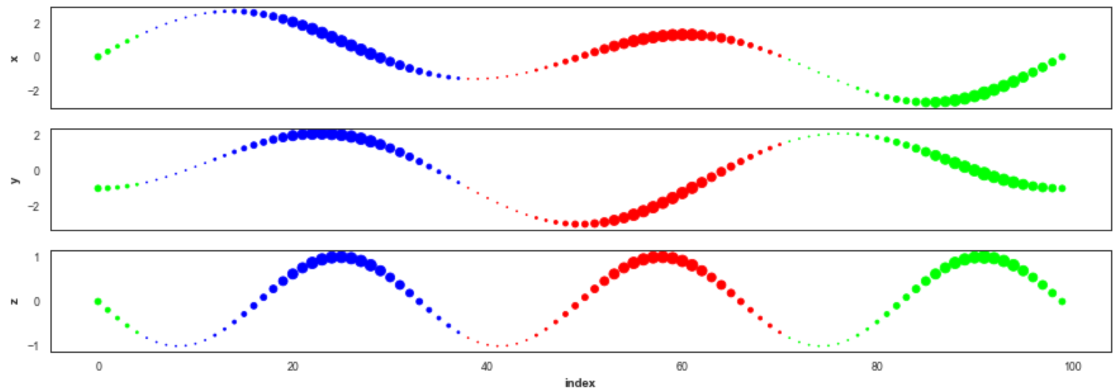


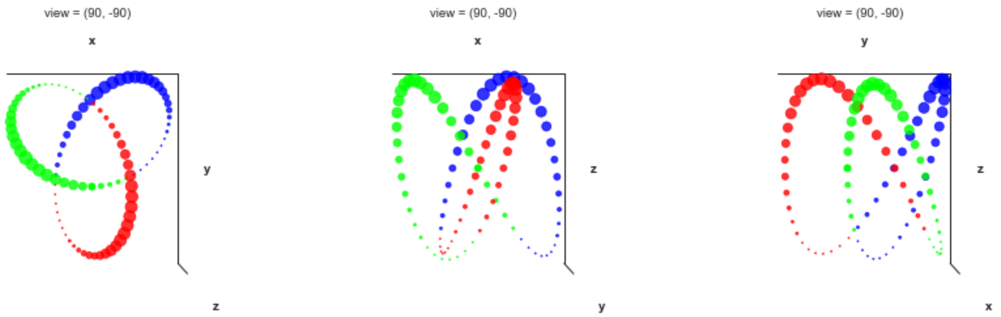

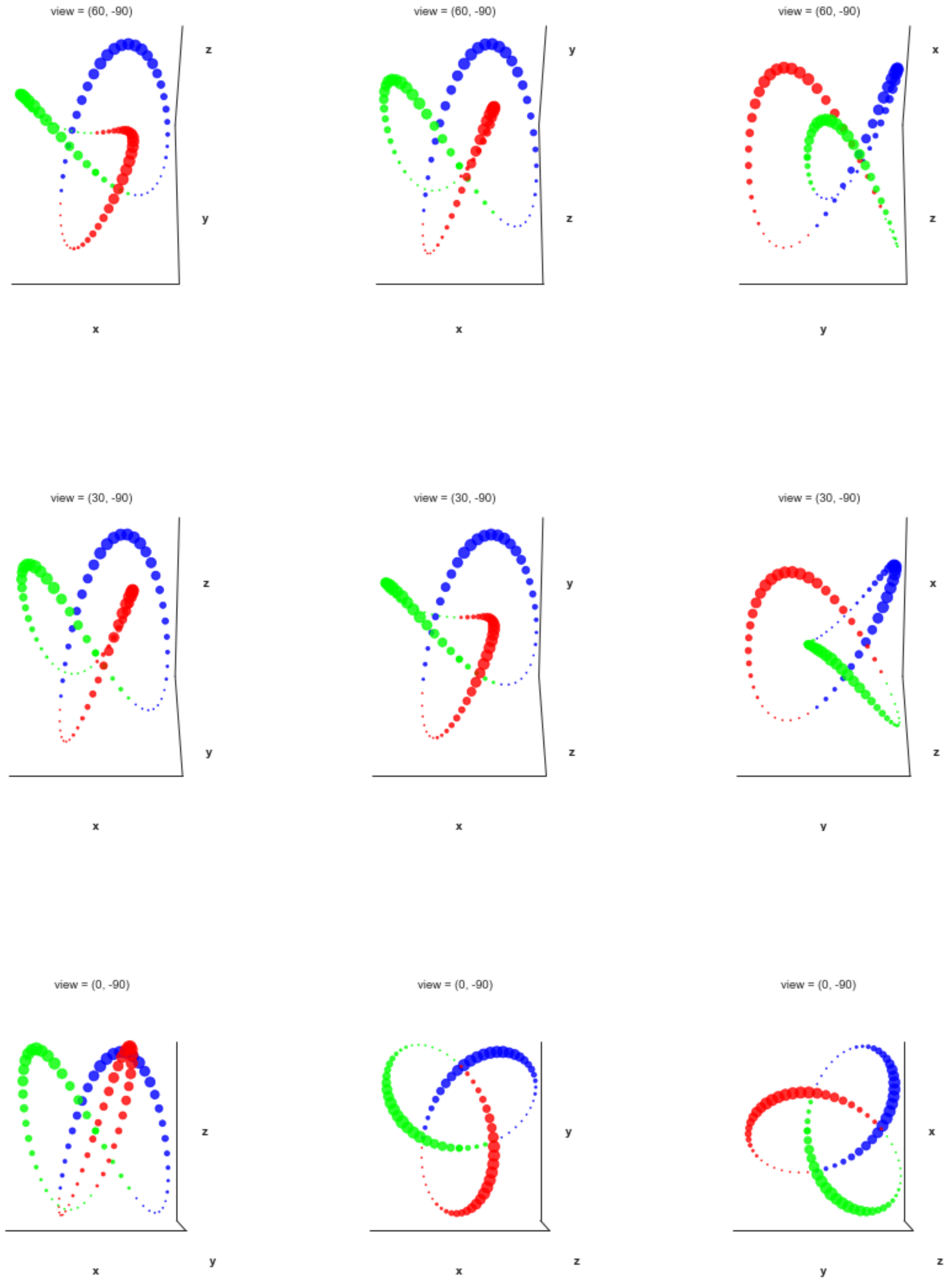


### 1.2.2 2.2 Visualize projections of the `data` using standard dimensionality reduction tools

In [9]:
```
pca = PCA(n_components=2)
x_pca = pca.fit_transform(X)
```

```
# visualize the projection
plt.scatter(x_pca[:, 0], x_pca[:, 1], c=data.coloring)
```

Out[9]: <matplotlib.collections.PathCollection at 0x1171b85c0>

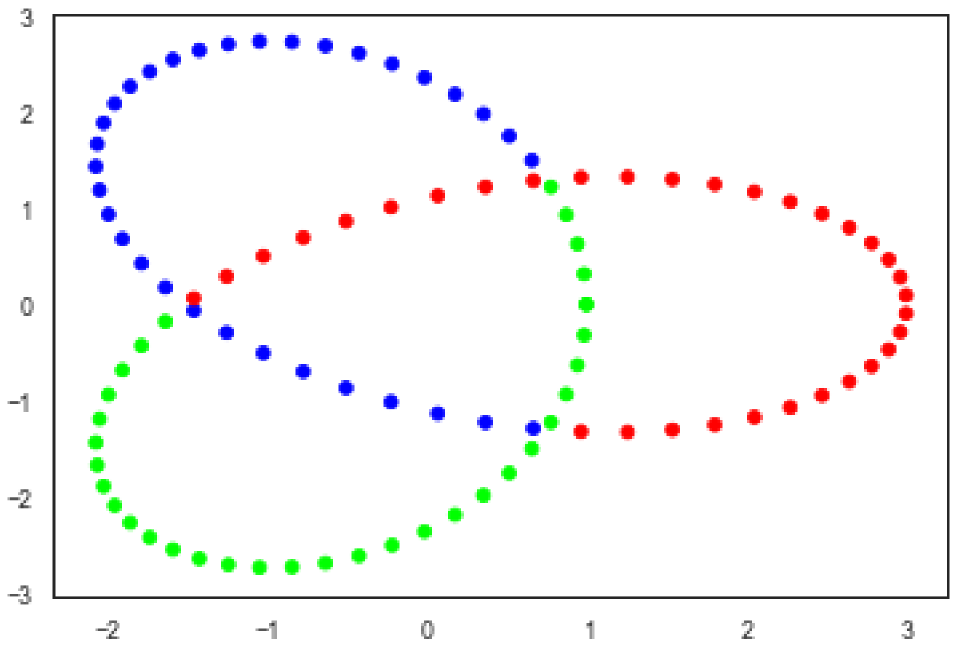


```
In [10]: tsne = TSNE(n_components=2, random_state=0)
         x_tsne = tsne.fit_transform(X)

         # visualize the projection
         plt.scatter(x_tsne[:, 0], x_tsne[:, 1], c=data.coloring)
```

Out[10]: <matplotlib.collections.PathCollection at 0x116ee0470>

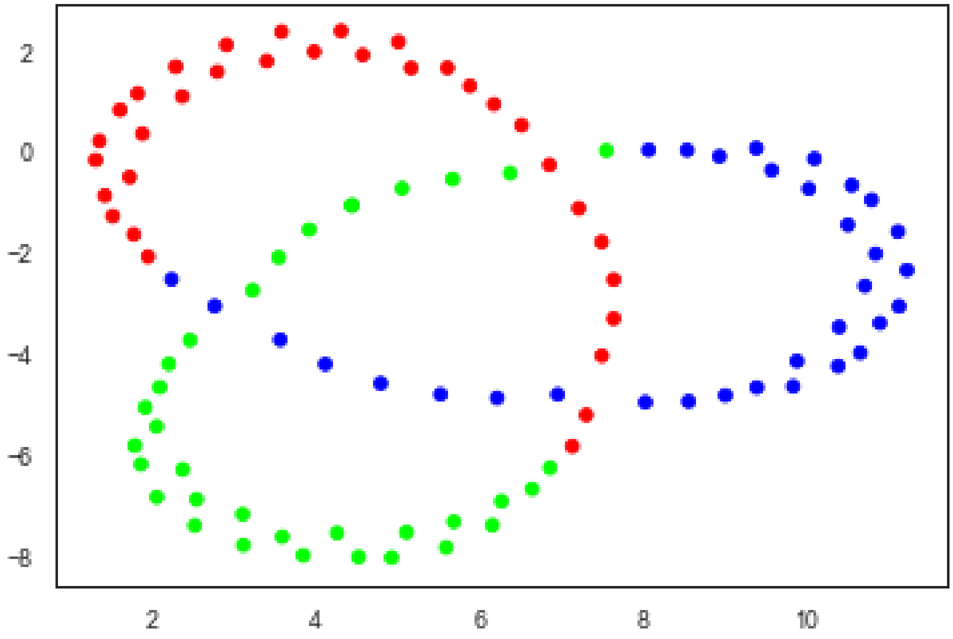


### 1.3 3 Generate a shape graph with kmapper

In [11]:
```
# init MAPPER
mapper = km.KeplerMapper(verbose=1)

# define the filter function (i.e., lens or projection)
projection = [0, 1]

# define the clusterer (i.e., for partial clustering in the original space)
clusterer = km.cluster.DBSCAN(eps=1)

# define the cover (i.e,. resolution=#bins, gain=%overlap)
cover = km.Cover(6, 0.8)
```

In [12]:
```
# run MAPPER
lens = mapper.fit_transform(X, projection=projection)
graph = mapper.map(lens, X_inverse, clusterer, coverer=cover)

# cache the parameters and results for later
mapped = Bunch(X=X, lens=lens, graph=graph, clusterer=clusterer, cover=cover)
```

```
..Composing projection pipeline length 1:
Projections: [0, 1]


Distance matrices: False
```

```
Scalers: MinMaxScaler(copy=True, feature_range=(0, 1))


..Projecting on data shaped (100, 3)

..Projecting data using: [0, 1]

..Scaling with: MinMaxScaler(copy=True, feature_range=(0, 1))

Mapping on data shaped (100, 3) using lens shaped (100, 2)

Creating 36 hypercubes.

Created 61 edges and 36 nodes in 0:00:00.031060.
```

#### 1.3.1 3.1 Visualize the `lens`

In [13]:
```
# ensure the lens is 2D
if len(lens.T) < 2:
    lens = np.c_[np.zeros_like(lens), lens]

# plot 1D view of lens
fig, axes = plt.subplots(1, 1, figsize=(8, 2))
for i,f in enumerate(lens.T):
    plt.scatter(np.arange(f.size), f, c=c, cmap='brg')

# plot 2D view of lens
fig, ax = plt.subplots(1, 1, figsize=(8, 8))
ax.scatter(*lens.T, c=c, s=100, alpha=0.8)
```

Out[13]: <matplotlib.collections.PathCollection at 0x116d8ce10>

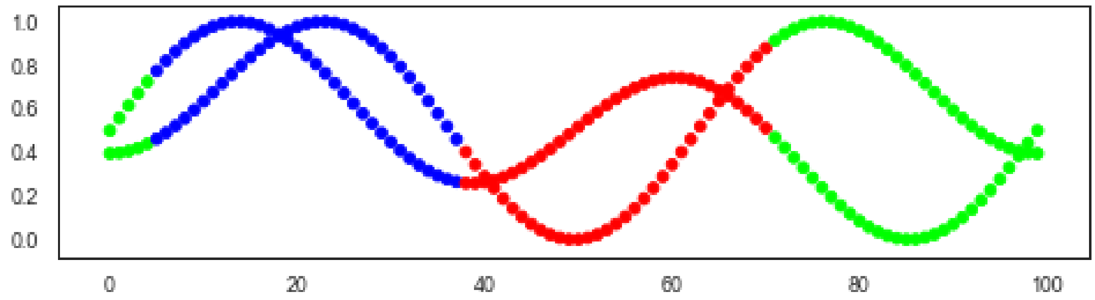

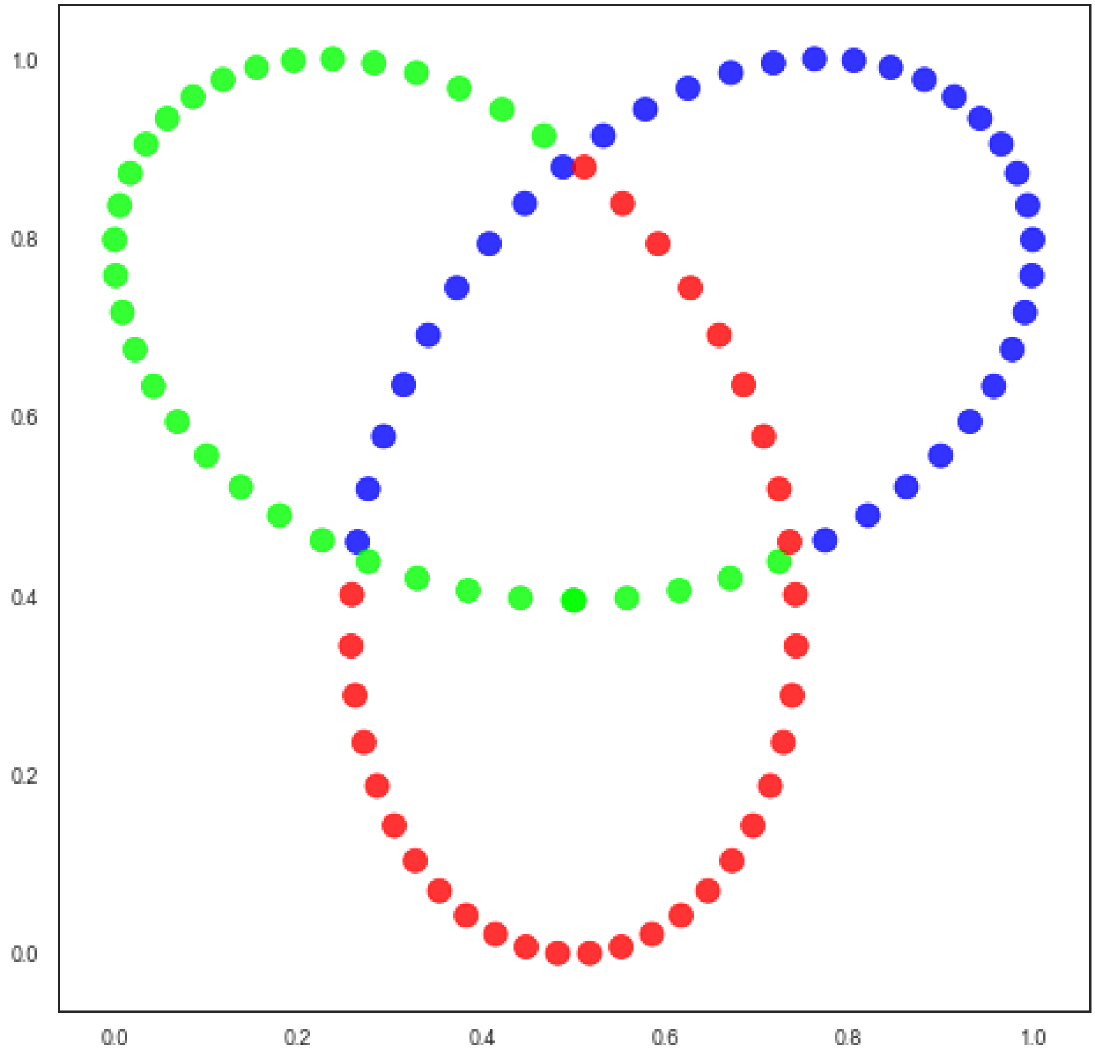


## 1.4 4 Visualize the shape graph with dyneusr

```
In [14]: # fit a DyNeuGraph to the shape graph
         dG = ds.DyNeuGraph(G=graph, y=y)

         # show shape of map_ (dimensions: nodes x members)
         print(dG.map_.shape)
```

```
<IPython.core.display.HTML object>


(100, 36)
```

### 1.4.1 4.1 Visualize the stages of the Mapper algorithm

```
In [15]: # draw MAPPER stages (and intermediates)
         _ = visualize_mapper_stages(data, dG=dG, **mapped, node_size=300, edge_color='gray')
```

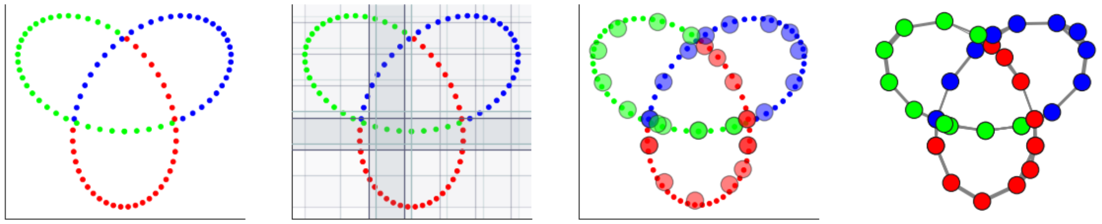

### 1.4.2 4.2 Visualize the shape graph with `networkx`

```
In [16]: # node color, size
         node_size = [100*len(y[_]) for n,_ in dG.G_.nodes(data='members')]
         node_color = [Counter(map(mpl.colors.to_hex, c[_])).most_common()[0][0] for n,_ in dG

         # edge color, size
         edge_size = [2*_ for u,v,_ in dG.G_.edges(data='size')]
         edge_sources = [dG.G_.nodes[u]['members'] for u,v in dG.G_.edges()]
         edge_targets = [dG.G_.nodes[v]['members'] for u,v in dG.G_.edges()]
         edge_color = None

         # plot nx
         fig, ax = plt.subplots(1, 1, figsize=(8,8))
         _ = ds.tools.networkx_utils.draw_nx(
             dG.G_, lens=lens, pos="inverse", layout=None, ax=ax,
             node_color=node_color, node_size=node_size,
             edge_color=edge_color, width=edge_size,
             )
```

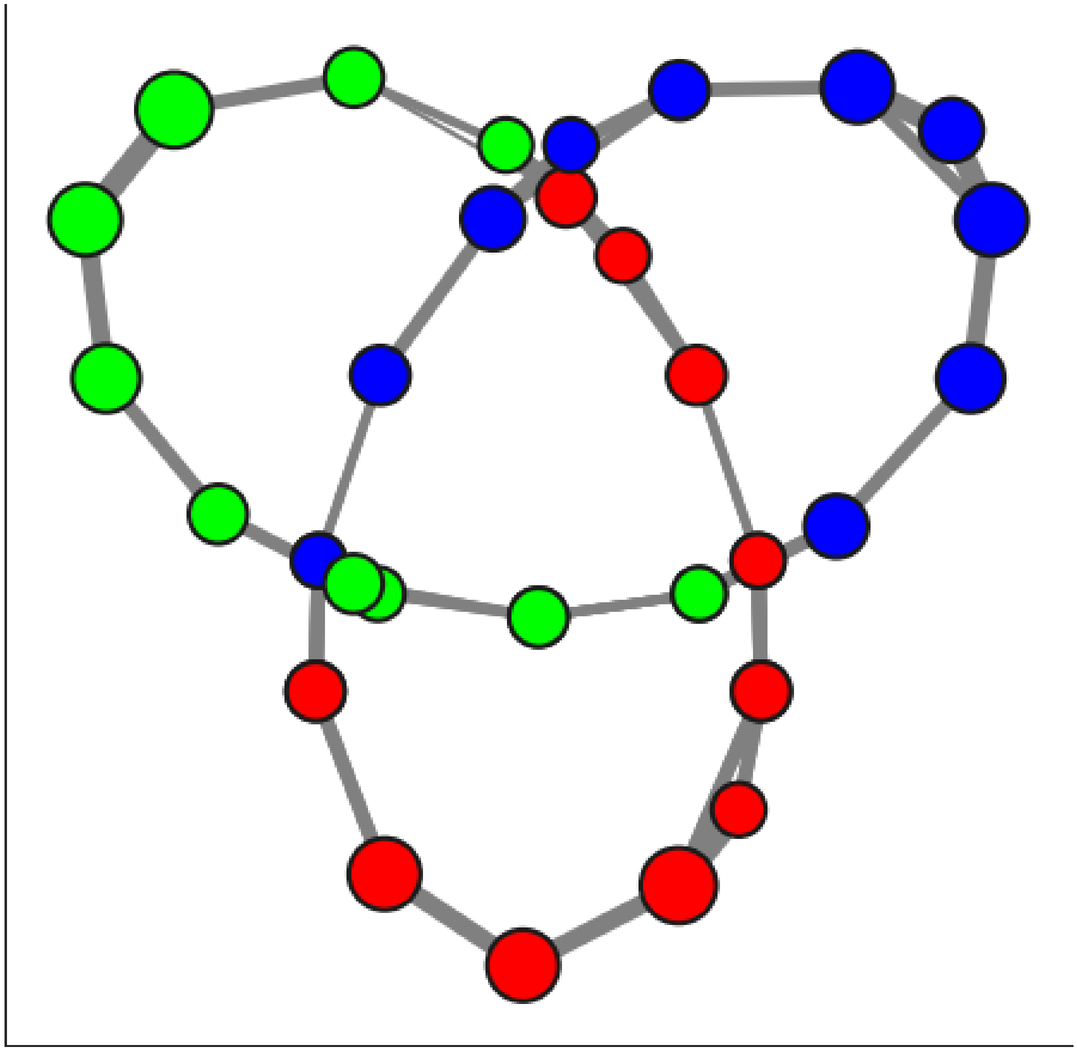

### 1.4.3 4.3 Visualize the shape graph with `networkx` (using the `kamada_kawai` layout)

```
In [17]: # node color, size
         node_size = [900 + 0*len(y[_]) for n,_ in dG.G_.nodes(data='members')]
         node_color = [Counter(map(mpl.colors.to_hex, c[_])).most_common()[0][0] for n,_ in dG

         # edge color, size
         edge_size = [2*_ for u,v,_ in dG.G_.edges(data='size')]
         edge_sources = [dG.G_.nodes[u]['members'] for u,v in dG.G_.edges()]
         edge_targets = [dG.G_.nodes[v]['members'] for u,v in dG.G_.edges()]
         edge_color = [Counter(map(mpl.colors.to_hex, c[s + t])).most_common()[0][0]
                       for s,t in zip(edge_sources, edge_targets)]

         # plot nx
```

```
fig, ax = plt.subplots(1, 1, figsize=(8,8))
_ = ds.tools.networkx_utils.draw_nx(
    dG.G_, lens=lens, pos=None, layout='kamada_kawai', ax=ax,
    node_color=node_color, node_size=node_size,
    edge_color=edge_color, width=edge_size,
    )
```

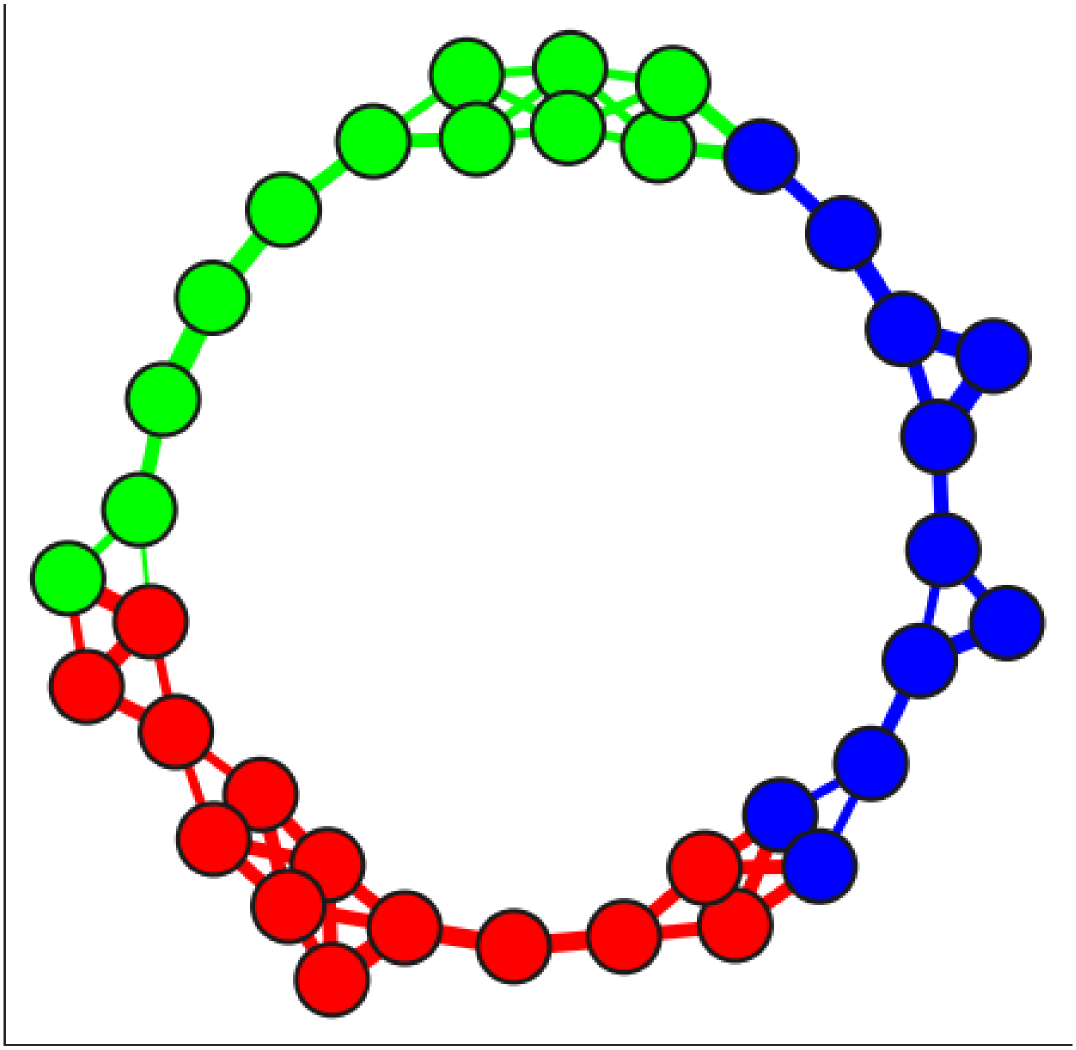

#### 1.4.4 4.4 Visualize the shape graph with dyneusr (using the d3-force layout)

```
In [18]: _ = dG.visualize('dyneusr_output_trefoil_knot.html', show=True, port=8801)
```

```
Already serving localhost:8801
[Force Graph] http://localhost:8801/dyneusr_output_trefoil_knot.html
```

```
<IPython.core.display.HTML object>
```

```
<IPython.lib.display.IFrame at 0x11b937c50>
```

```
<Figure size 432x288 with 0 Axes>
```
