# Supplementary material for: Generating dynamical neuroimaging spatiotemporal representations (DyNeuSR) using topological data analysis
Source: Netw Neurosci. 2019 Jul 1;3(3):763–78. doi: 10.1162/netn_a_00093 (PMC6663215; doi:10.1162/netn_a_00093)

Geniesse, C., Sporns, O., Petri, G., & Saggar, M. (2019). Supporting information for "Generating dynamical neuroimaging spatiotemporal representations (DyNeuSR) using topological data analysis." *Network Neuroscience*, *3*(3), 763–778. https://doi.org/10.1162/netn_a_00093

02_haxby_fmri

February 25, 2019

1 Example 2: Visualizing the Haxby (fMRI) dataset with `kmapper` + `dyneusr`

1.1 1 `import` libraries

In [1]:
```
# for auto-reloading external modules
%load_ext autoreload
%autoreload 2
```

In [2]:
```
import os
```

In [3]:
```
import numpy as np
import pandas as pd
import scipy as sp

import networkx as nx
from collections import Counter
```

In [4]:
```
import matplotlib as mpl
import matplotlib.pyplot as plt
import seaborn as sns
sns.set("paper", "white")

%matplotlib inline
```

1.1.1 1.1 `import kmapper`

Here, we will use the KeplerMapper (`kmapper`) implementation of the Mapper algorithm.

We will also import sklearn implementations of PCA and TSNE to use as (linear and non-linear) filter functions for Mapper.

In [5]:
```
import kmapper as km
from sklearn.manifold import TSNE
from sklearn.decomposition import PCA
```

1.1.2 1.2 import dyneusr

Note, dyneusr provides a wrapper around kmapper with support for caching each step of the Mapper algorithm. This will speed things up, especially when generating several shape graphs for different subjects. For a more detailed walk-through of how to generate a shape graph using kmapper, see 01_trefoil_knot/01_trefoil_knot.ipynb.

```
In [6]: import dyneusr as ds

        # kmapper wrappers
        from dyneusr.mapper.wrappers import KMapperWrapper
        from dyneusr.mapper.utils import optimize_cover, optimize_dbscan
```

1.2 2 Load data

```
In [7]: from load_data import load_haxby, Bunch
```

Here, we actually use nilearn.datasets.fetch_haxby to fetch the files for the Haxby dataset. To make this note book more readable, we wrapped several steps into a single load_haxby function, stored in load_data.py.

These steps include: 1. fetching the Haxby dataset files; 2. performing some preprocessing; and 3. storing the data for each subject as an sklearn.datasets.base.Bunch object for easy access.

A list of these objects, one for each subject, is stored in a higher-level object, which also stores some other important meta-data shared across subjects. This higher-level object is returned by the function, load_haxby.

```
In [8]: # load data for all 6 subjects, but only sessions 4-5
        haxby = load_haxby(subjects=-1, sessions=[4,5], targets=None)

        # extract subjects, print some information about them
        subjects = haxby.subjects
        for i, subject in enumerate(subjects):
            print(i, subject.name, subject.data.shape, subject.target.shape)
```

```
pixdim[0] (qfac) should be 1 (default) or -1; setting qfac to 1
INFO:nibabel.global:pixdim[0] (qfac) should be 1 (default) or -1; setting qfac to 1
pixdim[0] (qfac) should be 1 (default) or -1; setting qfac to 1
INFO:nibabel.global:pixdim[0] (qfac) should be 1 (default) or -1; setting qfac to 1
pixdim[0] (qfac) should be 1 (default) or -1; setting qfac to 1
INFO:nibabel.global:pixdim[0] (qfac) should be 1 (default) or -1; setting qfac to 1
pixdim[0] (qfac) should be 1 (default) or -1; setting qfac to 1
INFO:nibabel.global:pixdim[0] (qfac) should be 1 (default) or -1; setting qfac to 1
pixdim[0] (qfac) should be 1 (default) or -1; setting qfac to 1
INFO:nibabel.global:pixdim[0] (qfac) should be 1 (default) or -1; setting qfac to 1
pixdim[0] (qfac) should be 1 (default) or -1; setting qfac to 1
INFO:nibabel.global:pixdim[0] (qfac) should be 1 (default) or -1; setting qfac to 1
```

```
0 subj1_sess4_5 (242, 577) (242, 9)
1 subj2_sess4_5 (242, 464) (242, 9)
2 subj3_sess4_5 (242, 307) (242, 9)
3 subj4_sess4_5 (242, 675) (242, 9)
4 subj5_sess4_5 (242, 422) (242, 9)
5 subj6_sess4_5 (242, 348) (242, 9)
```

1.2.1 2.1 Visualize the data

```
In [9]: # inspect the first 10 voxels
        for subject in haxby.subjects:
            _ = subject.data.iloc[:,:10].plot(
                figsize=(20,2),
                legend=False,
                title=subject.name
                )
```

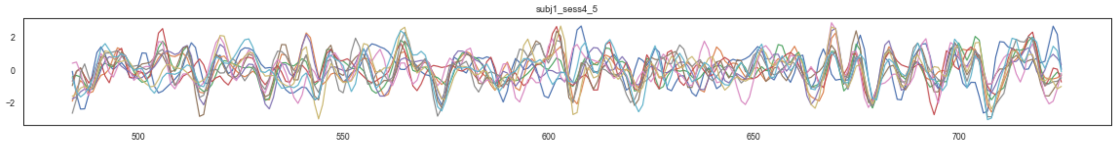

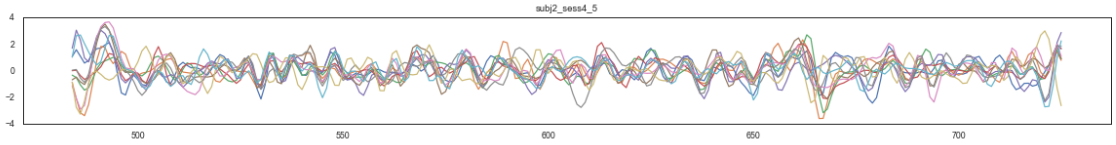

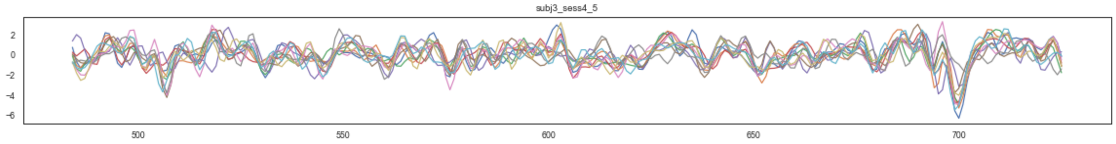

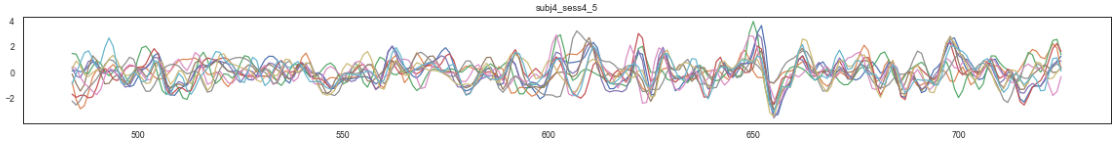

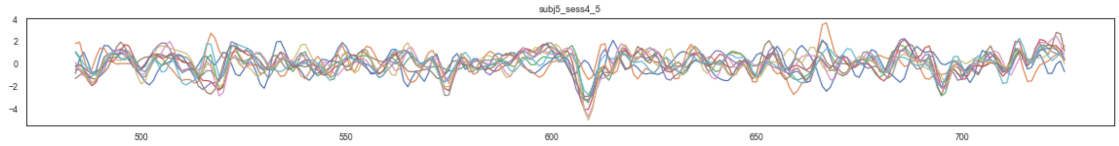

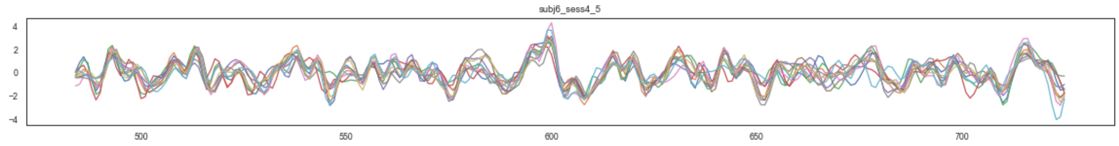

1.2.2 2.2 Visualize the meta data

In [10]:
```
# inspect the first 10 voxels
for subject in haxby.subjects:
    _ = subject.meta.iloc[:,:10].plot(
        figsize=(20,2),
        legend=False,
        title=subject.name
        )
```

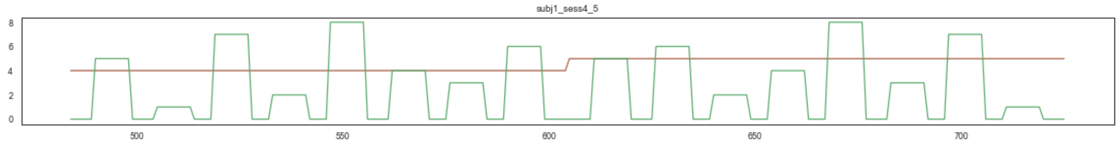

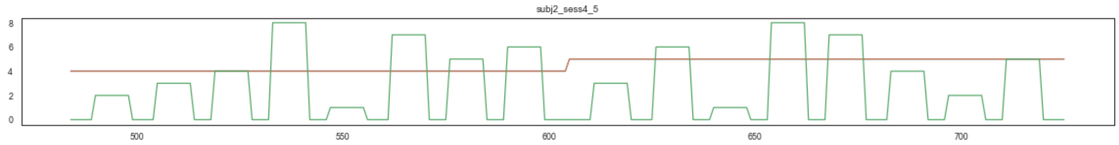

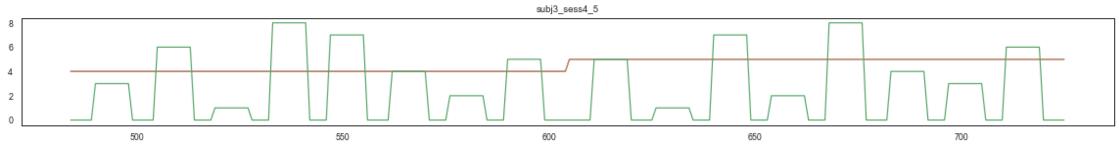

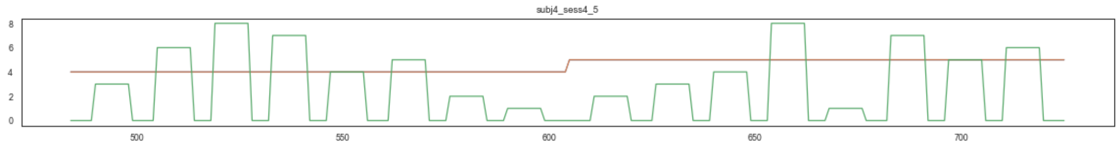

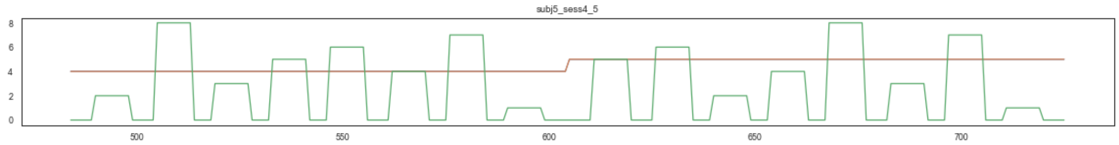

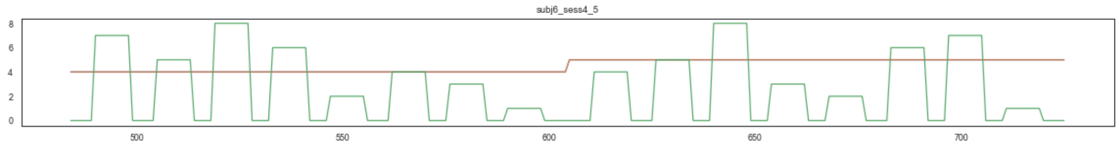

1.3 3 Generate a shape graph for each subject with kmapper

In [11]:
```
# loop over sessions, run for each
for i, subject in enumerate(subjects):

    ### Setup: extract X, y for each subject
    X = subject.data.values.copy()
    y = subject.target.copy()
    y = y.reindex(columns=haxby.target_names)
    print('[subject {}]'.format(subject.name))
    print(' X has shape: {}'.format(X.shape))
    print(' y has shape: {}'.format(y.shape))


    ### Run kmapper
    print("\nGenerating shape graph...")
    projection = TSNE(
        perplexity=50, random_state=0,
        init='pca', method='exact'
        )
    clusterer = optimize_dbscan(X)
    cover = optimize_cover(
        X, r=15, g=0.67,
        scale_r=not True,
```

```
        scale_limits=True
        )
    print('\n projection = {}'.format(projection))
    print('\n clusterer = {}'.format(clusterer))
    print('\n cover = {}'.format(cover.__dict__))

    # run kmapper
    mapper = KMapperWrapper(
        memory='dyneusr_cache/{}'.format(subject.name)
        )
    mapper.fit_lens(X, projection=projection)
    mapper.fit_graph(data=X, clusterer=clusterer, cover=cover)

    # extract results
    lens = mapper.lens_.copy()
    graph = dict(mapper.graph_)


    ### Fit DyNeuGraph
    print("\nProcessing shape graph...")
    dG = ds.DyNeuGraph(
        G=graph, y=y,
        labels=haxby.target_names,
        colors=haxby.target_colors,
        cmap=haxby.cmap
        )

    # store data
    subject.mapped = Bunch(
        data=X,
        lens=lens,
        graph=graph,
        dG=dG, X=X, y=y
        )
    subjects[i] = subject
    print()
    print()
```

```
[subject subj1_sess4_5]
 X has shape: (242, 577)
 y has shape: (242, 9)

Generating shape graph...

 projection = TSNE(angle=0.5, early_exaggeration=12.0, init='pca', learning_rate=200.0,
   method='exact', metric='euclidean', min_grad_norm=1e-07, n_components=2,
   n_iter=1000, n_iter_without_progress=300, perplexity=50, random_state=0,
   verbose=0)
```

```
 clusterer = DBSCAN(algorithm='auto', eps=26.3271381223901, leaf_size=15,
    metric='minkowski', metric_params=None, min_samples=2, n_jobs=1, p=2)

 cover = {'n_cubes': 17, 'perc_overlap': 0.67, 'limits': array([[-0.04466667,  1.04466667],
       [-0.04466667,  1.04466667]])}

Processing shape graph...


<IPython.core.display.HTML object>




[subject subj2_sess4_5]
 X has shape: (242, 464)
 y has shape: (242, 9)

Generating shape graph...

 projection = TSNE(angle=0.5, early_exaggeration=12.0, init='pca', learning_rate=200.0,
   method='exact', metric='euclidean', min_grad_norm=1e-07, n_components=2,
   n_iter=1000, n_iter_without_progress=300, perplexity=50, random_state=0,
   verbose=0)

 clusterer = DBSCAN(algorithm='auto', eps=27.605001592992302, leaf_size=15,
    metric='minkowski', metric_params=None, min_samples=2, n_jobs=1, p=2)

 cover = {'n_cubes': 17, 'perc_overlap': 0.67, 'limits': array([[-0.04466667,  1.04466667],
       [-0.04466667,  1.04466667]])}

Processing shape graph...


<IPython.core.display.HTML object>




[subject subj3_sess4_5]
 X has shape: (242, 307)
 y has shape: (242, 9)

Generating shape graph...

 projection = TSNE(angle=0.5, early_exaggeration=12.0, init='pca', learning_rate=200.0,
   method='exact', metric='euclidean', min_grad_norm=1e-07, n_components=2,
   n_iter=1000, n_iter_without_progress=300, perplexity=50, random_state=0,
```

```
   verbose=0)

 clusterer = DBSCAN(algorithm='auto', eps=24.668255970446275, leaf_size=15,
    metric='minkowski', metric_params=None, min_samples=2, n_jobs=1, p=2)

 cover = {'n_cubes': 17, 'perc_overlap': 0.67, 'limits': array([[-0.04466667,  1.04466667],
       [-0.04466667,  1.04466667]])}

Processing shape graph...


<IPython.core.display.HTML object>




[subject subj4_sess4_5]
 X has shape: (242, 675)
 y has shape: (242, 9)

Generating shape graph...

 projection = TSNE(angle=0.5, early_exaggeration=12.0, init='pca', learning_rate=200.0,
   method='exact', metric='euclidean', min_grad_norm=1e-07, n_components=2,
   n_iter=1000, n_iter_without_progress=300, perplexity=50, random_state=0,
   verbose=0)

 clusterer = DBSCAN(algorithm='auto', eps=28.004985102867675, leaf_size=15,
    metric='minkowski', metric_params=None, min_samples=2, n_jobs=1, p=2)

 cover = {'n_cubes': 17, 'perc_overlap': 0.67, 'limits': array([[-0.04466667,  1.04466667],
       [-0.04466667,  1.04466667]])}

Processing shape graph...


<IPython.core.display.HTML object>




[subject subj5_sess4_5]
 X has shape: (242, 422)
 y has shape: (242, 9)

Generating shape graph...

 projection = TSNE(angle=0.5, early_exaggeration=12.0, init='pca', learning_rate=200.0,
   method='exact', metric='euclidean', min_grad_norm=1e-07, n_components=2,
```

```
   n_iter=1000, n_iter_without_progress=300, perplexity=50, random_state=0,
   verbose=0)

 clusterer = DBSCAN(algorithm='auto', eps=23.616781132201332, leaf_size=15,
    metric='minkowski', metric_params=None, min_samples=2, n_jobs=1, p=2)

 cover = {'n_cubes': 17, 'perc_overlap': 0.67, 'limits': array([[-0.04466667,  1.04466667],
       [-0.04466667,  1.04466667]])}

Processing shape graph...


<IPython.core.display.HTML object>




[subject subj6_sess4_5]
 X has shape: (242, 348)
 y has shape: (242, 9)

Generating shape graph...

 projection = TSNE(angle=0.5, early_exaggeration=12.0, init='pca', learning_rate=200.0,
   method='exact', metric='euclidean', min_grad_norm=1e-07, n_components=2,
   n_iter=1000, n_iter_without_progress=300, perplexity=50, random_state=0,
   verbose=0)

 clusterer = DBSCAN(algorithm='auto', eps=20.11123226958869, leaf_size=15,
    metric='minkowski', metric_params=None, min_samples=2, n_jobs=1, p=2)

 cover = {'n_cubes': 17, 'perc_overlap': 0.67, 'limits': array([[-0.04466667,  1.04466667],
       [-0.04466667,  1.04466667]])}

Processing shape graph...


<IPython.core.display.HTML object>
```

1.4 4 Visualize and annotate the shape graph with dyneusr

```
In [12]: # select subject to inspect below
         subject = subjects[0]
```

```
# copy inputs to Mapper
data = subject.data.copy()
meta = subject.meta.copy()

# copy outputs from Mapper
X = subject.mapped.X.copy()
y = subject.mapped.y.copy()
lens = subject.mapped.lens.copy()
dG = subject.mapped.dG
```

1.4.1 4.1 Visualize the lens

In [13]:
```
### View some quick results (e.g., the lens)
fig, axes = plt.subplots(2, 4, figsize=(16, 8))

# loop over axis for each category of visual stimuli
c_cols = [_ for _ in y.columns if _ not in ['rest']]
for ax, stim in zip(np.ravel(axes), c_cols):

    # mask the stimuli for this category
    y_mask = (y[stim] == 1)

    # plot the lens, coloring only the category == stim
    ax.scatter(*lens.T,
               c=y[stim], cmap='winter')
    ax.scatter(*lens[y_mask].T,
               c=y[y_mask][stim], cmap='winter_r')
    ax.set_title(stim, fontsize=24)
    ax.axis('off')
```

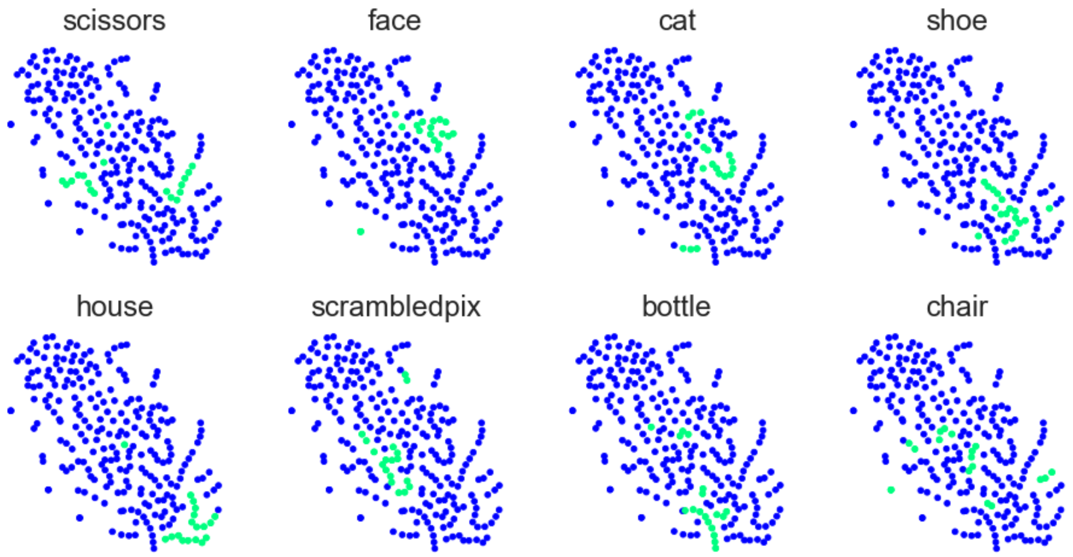

1.4.2 4.2 Visualize the stages of Mapper

In [14]:
```
# we need to set y to a coloring
subject.y = subject.meta.target.values.copy()

# draw Mapper stages (and intermediates)
_ = ds.tools.networkx_utils.visualize_mapper_stages(
    data=subject, lens=lens, cover=cover, graph=graph,dG=dG,
    node_size=10,
    )
```

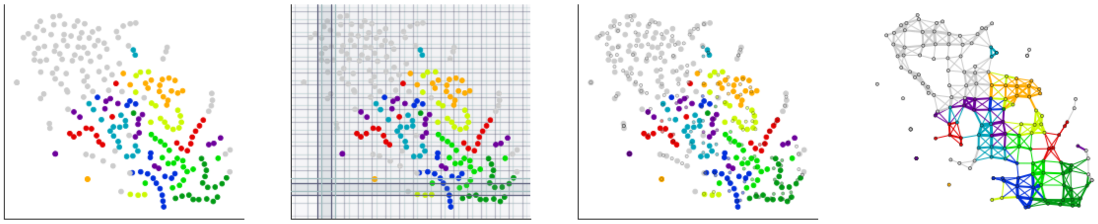

1.4.3 4.3 Visualize the shape graph with dyneusr

In [15]:
```
dG.visualize(
    'dyneusr_output_haxby_{}.html'.format(subject.name),
    show=True, port=8802
    )
```

```
Already serving localhost:8802
[Force Graph] http://localhost:8802/dyneusr_output_haxby_subj1_sess4_5.html


<IPython.core.display.HTML object>


<IPython.lib.display.IFrame at 0x11e2c57f0>
```

Out[15]:
```
DyNeuGraph()
```

```
<Figure size 432x288 with 0 Axes>
```

1.4.4 4.4 Qualitative analysis

In [16]:
```python
# define TR x Node, TR x TR, and TR x Stimuli matrices
TxN = dG.M.copy()
TxT = dG.TCM.copy()
TxS = y.copy().iloc[:, 1:]

# Compute Node x Stimuli, Stimuli x Stimuli
NxS = TxN.T @ TxS
SxS = NxS.T @ NxS
SxS /= SxS.max()


# plot the heatmaps
fig,axes = plt.subplots(1, 3, figsize=(12, 3))
sns.heatmap(TxT, ax=axes[0], cmap='jet')
sns.heatmap(TxS, ax=axes[1], cmap='jet', xticklabels=TxS.columns)
sns.heatmap(NxS, ax=axes[2], cmap='jet', xticklabels=TxS.columns)


# plot the heatmap of category similarity
fig,axes = plt.subplots(1, 1, figsize=(15, 12))
axes = np.ravel(axes)
sns.heatmap(SxS, ax=axes[0], cmap='jet',
            xticklabels=TxS.columns, yticklabels=TxS.columns)
```

Out[16]:
```
<matplotlib.axes._subplots.AxesSubplot at 0x120c86978>
```

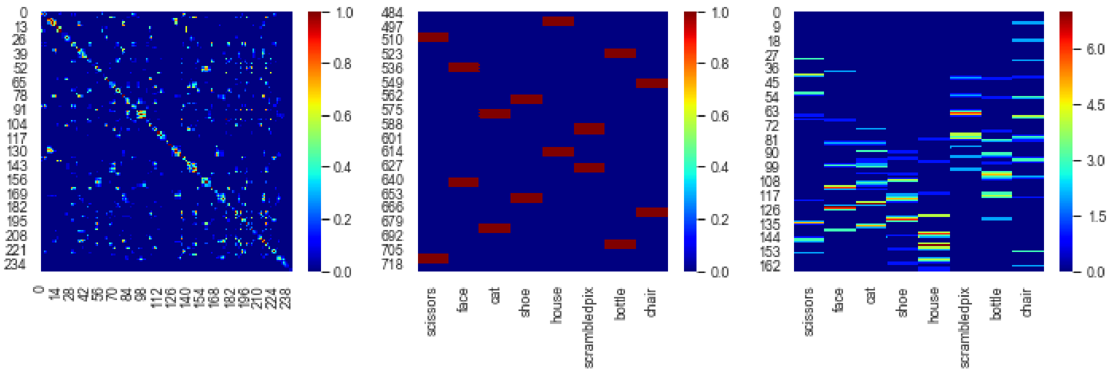

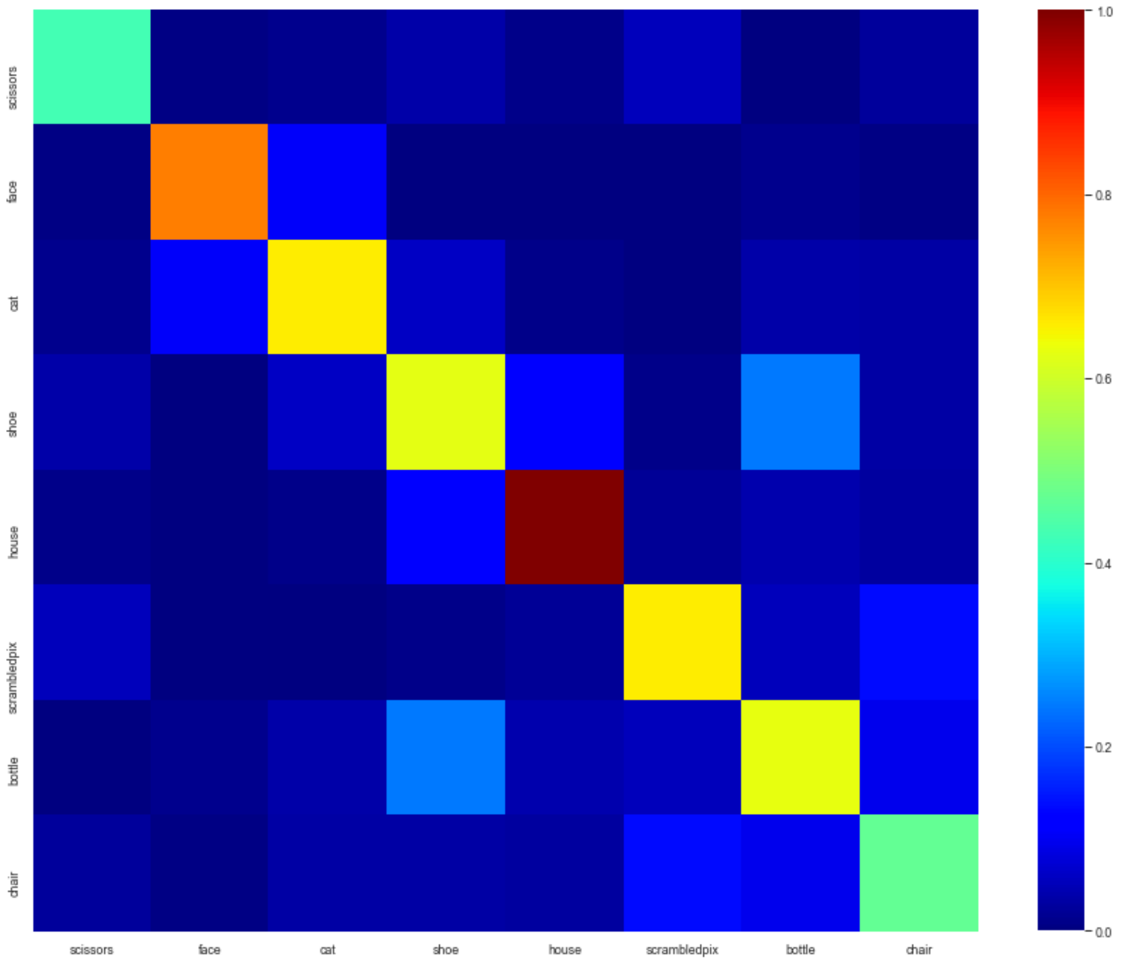

1.5 5 Anchor the shape graph to brain anatomy with dyneusr

1.5.1 5.1 Estimate average brain images for each time frame

In [17]:
```
# get mixtures (time frames to average over)
mixtures = dG.mixtures_

# estimate and plot average brain images
brain_images = ds.tools.mixture.simple_mixtures(
    subject, mixtures=mixtures,
    save_dir='tooltips_{}'.format(subject.name),
    prefix='TR_', targets=subject.meta.labels.tolist(),
    show_every_n=12, print_every_n=0,
    threshold=None, fwhm=None,
    kind='mean', mode='glass', figsize=(4, 4),
    plot_kws=dict(
        display_mode='z', cut_coords=1,
        vmin=-3, vmax=3, threshold=0, colorbar=True,
```

```
    ),
)
```

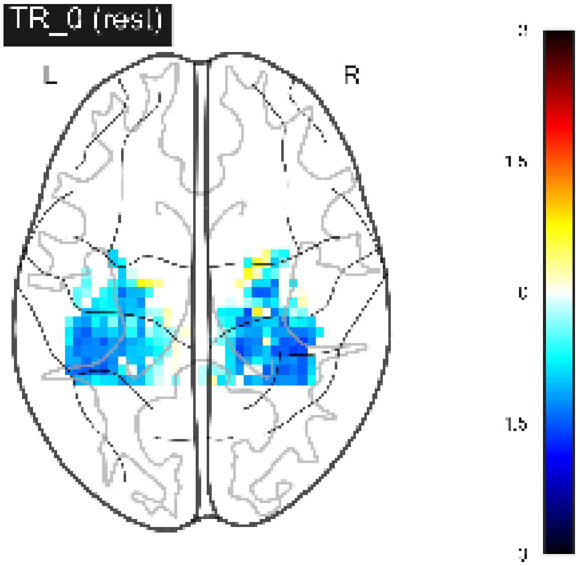
TR_0 (rest)
L
R

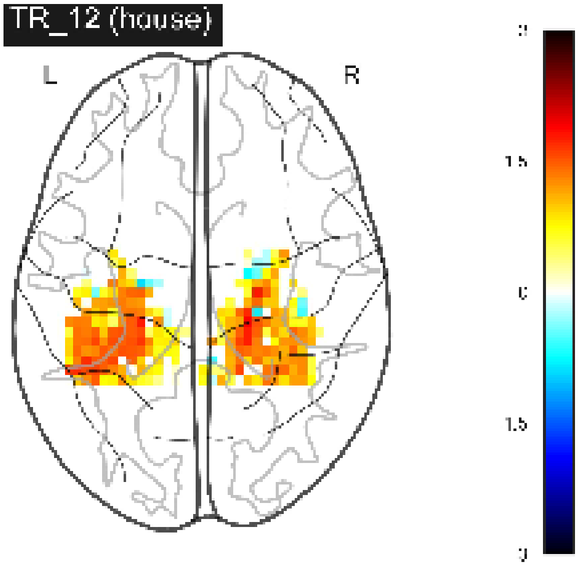
TR_12 (house)
L
R

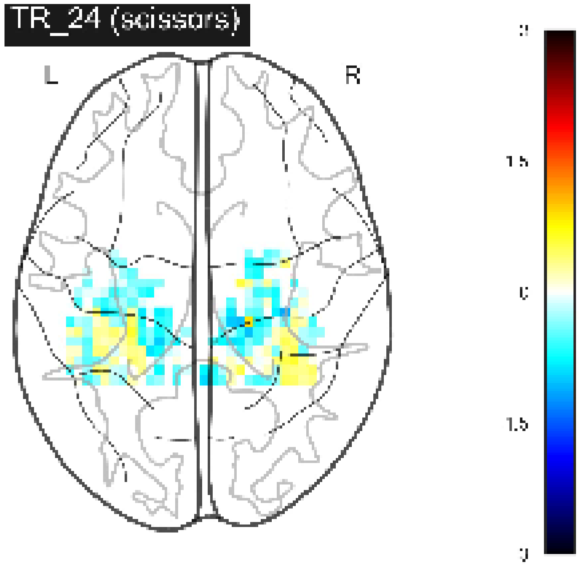
TR_24 (scissors)
L
R

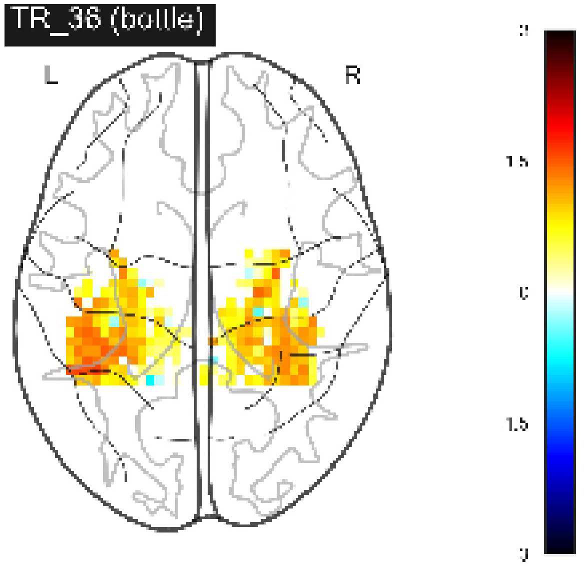
TR_36 (bottle)
L
R

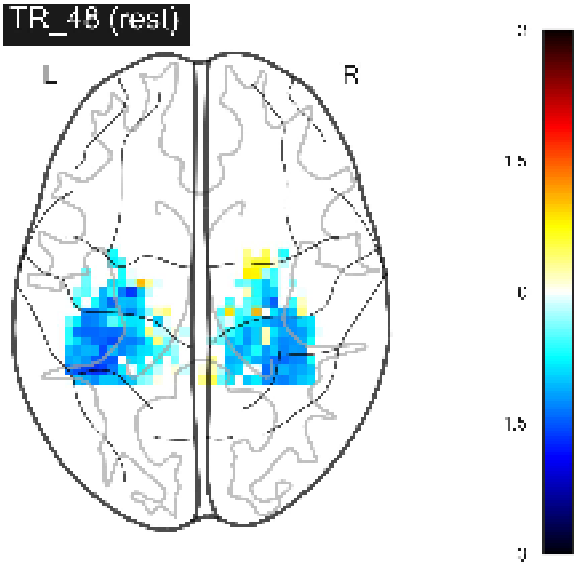
TR_48 (resl)
L
R

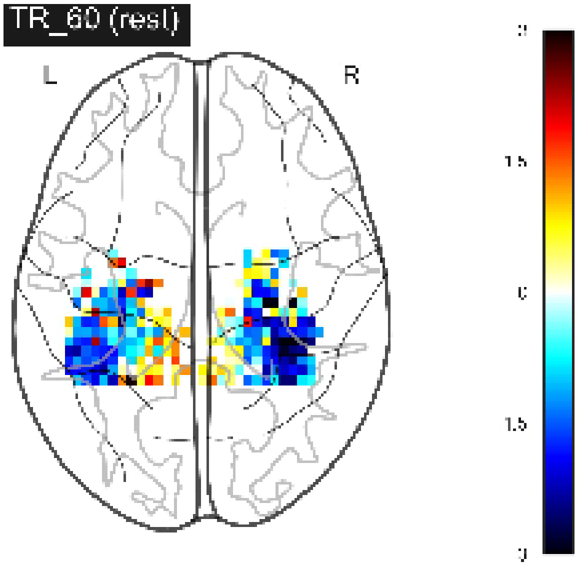
TR_60 (resl)
L
R

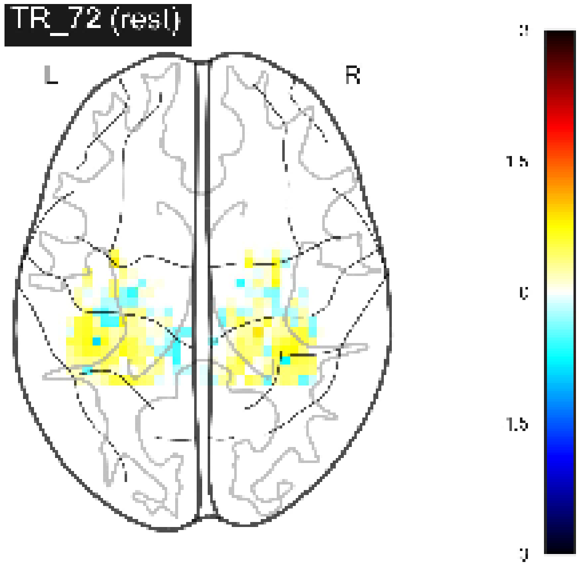
TR_72 (rest)
L
R

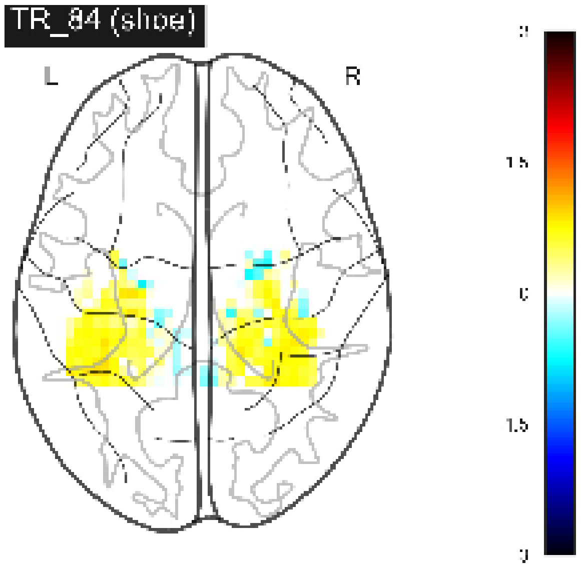
TR_84 (shoe)
L
R

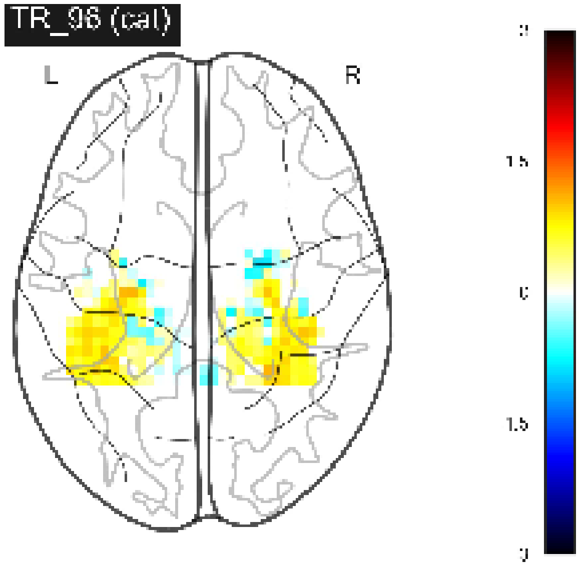
TR_98 (cat)
L
R

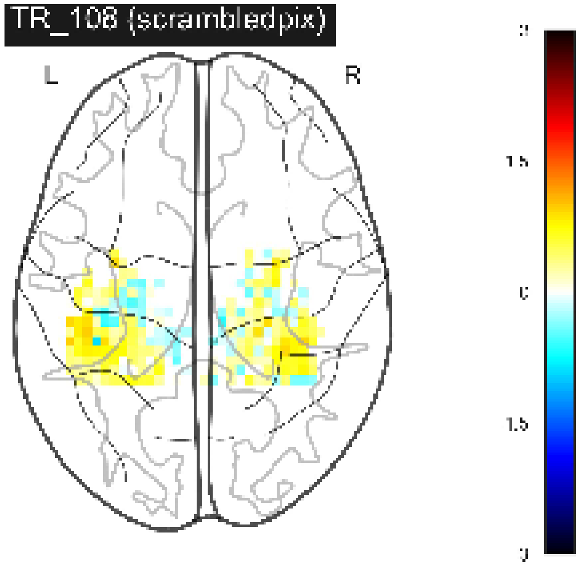
TR_108 (scrambledpix)
L
R

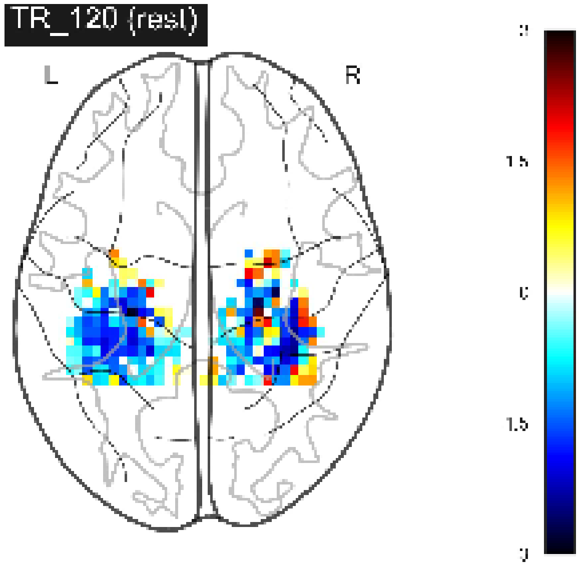
TR_120 (rest)
L
R

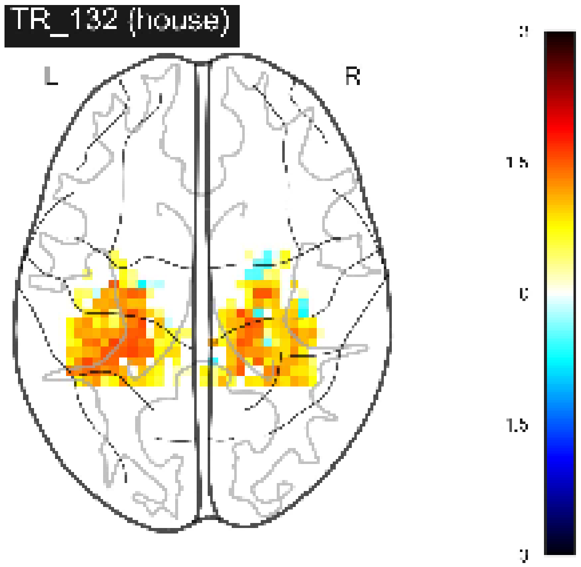
TR_132 (house)
L
R

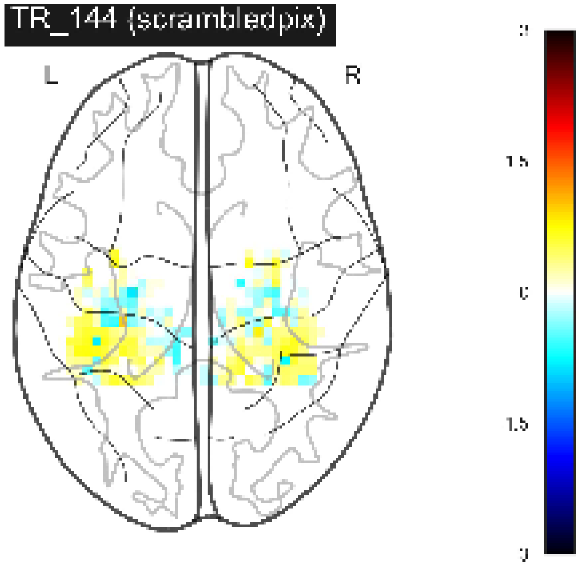
TR_144 (scrambledpix)
L
R

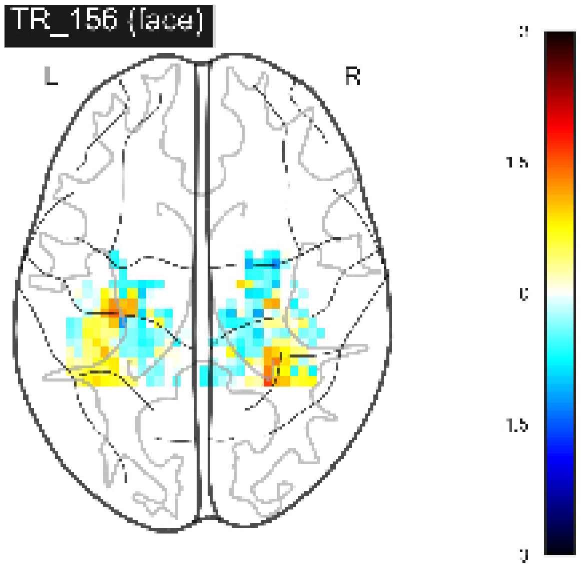
TR_156 (face)
L
R

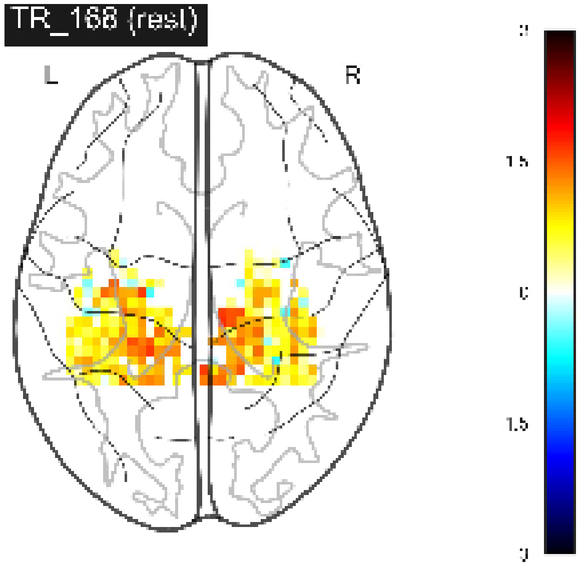
TR_168 (rest)
L
R
1.5
0
1.5

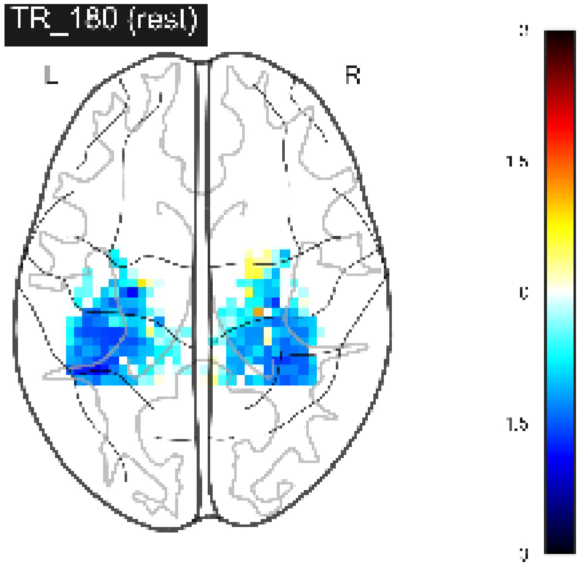
TR_180 (rest)
L
R
1.5
0
1.5

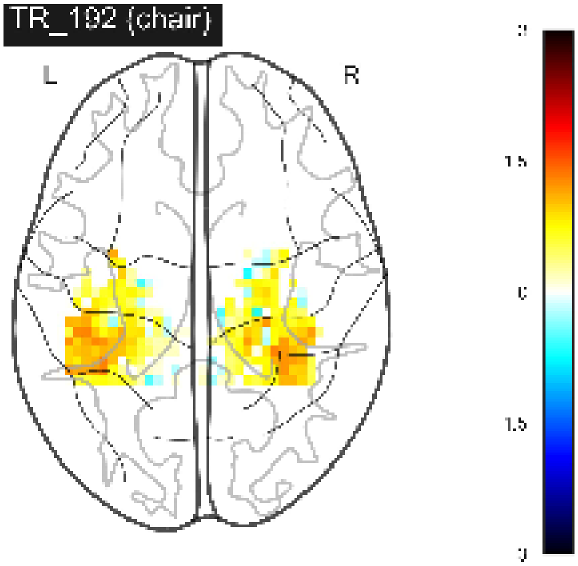
TR_192 (chair)
L
R

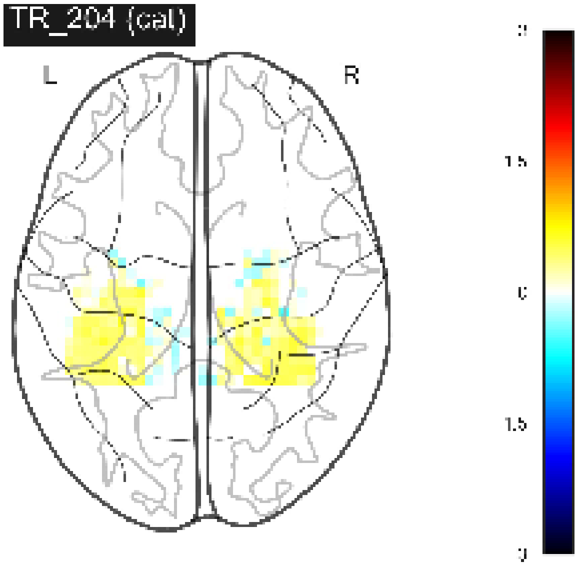
TR_204 (cat)
L
R

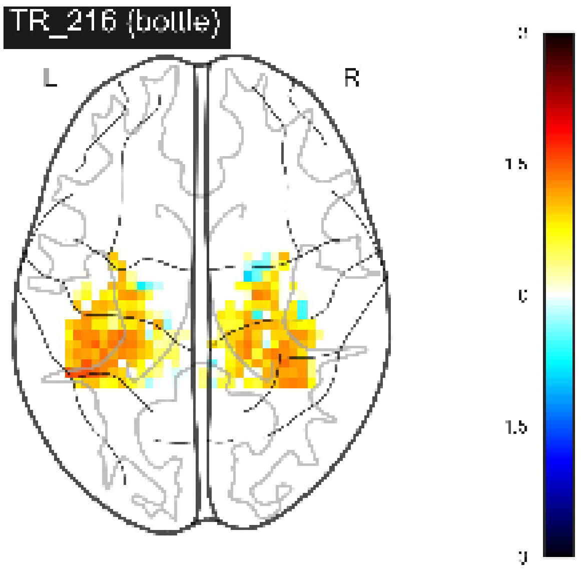
TR_216 (bottle)
L
R

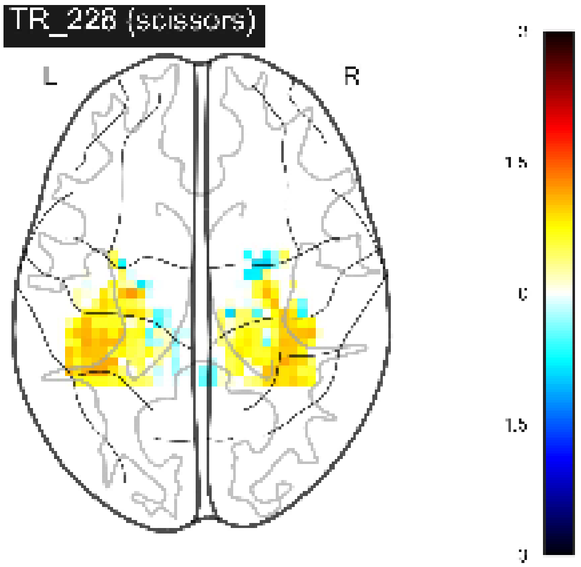
TR_228 (scissors)
L
R

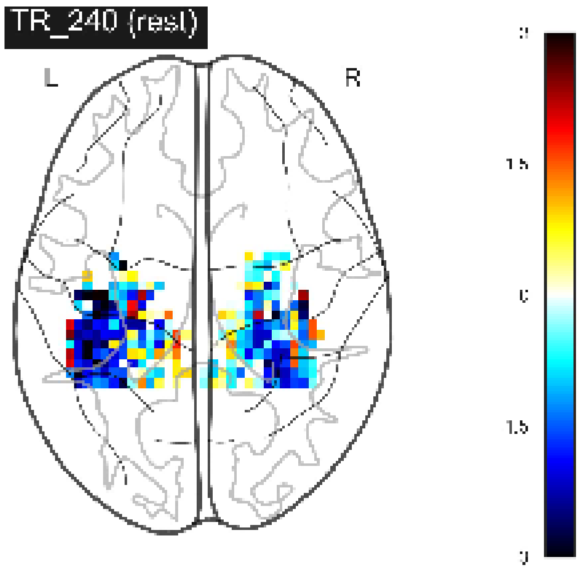

[done]

1.5.2 5.2 Annotate nodes in the shape graph with the brain images

```
In [18]: _ = dG.annotate(image=brain_images)
```

1.5.3 5.3 Visualize the shape graph with dyneusr (using the d3-force layout)

```
In [19]: dG.visualize(
             'dyneusr_output_haxby_anat_{}.html'.format(subject.name),
             show=True, port=8802
             )
```

```
Already serving localhost:8802
[Force Graph] http://localhost:8802/dyneusr_output_haxby_anat_subj1_sess4_5.html


<IPython.core.display.HTML object>


<IPython.lib.display.IFrame at 0x120a7c6d8>
```

```
Out[19]: DyNeuGraph()
```

```
<Figure size 432x288 with 0 Axes>
```

1.6 6 Capture temporal transitions in brain activity with dyneusr

In [20]:
```
# aggregate the TCMs into a single matrix
TCMs = np.array([_.mapped.dG.TCM.copy() for _ in subjects])
print("TCMs has shape:", TCMs.shape)
```

```
TCMs has shape: (6, 242, 242)
```

1.6.1 6.1 Visualize the temporal connectivity matrix (TCM)

In [21]:
```
sns.heatmap(dG.TCM, cbar=True, cmap='jet', figure=plt.figure(figsize=(12,10)))
plt.title('{}'.format(subject.name), fontsize=24, fontweight='bold')
plt.xlabel('Time frame (TR)', fontsize=18)
plt.ylabel('Time frame (TR)', fontsize=18)
```

Out[21]:
```
Text(86.7,0.5,'Time frame (TR)')
```

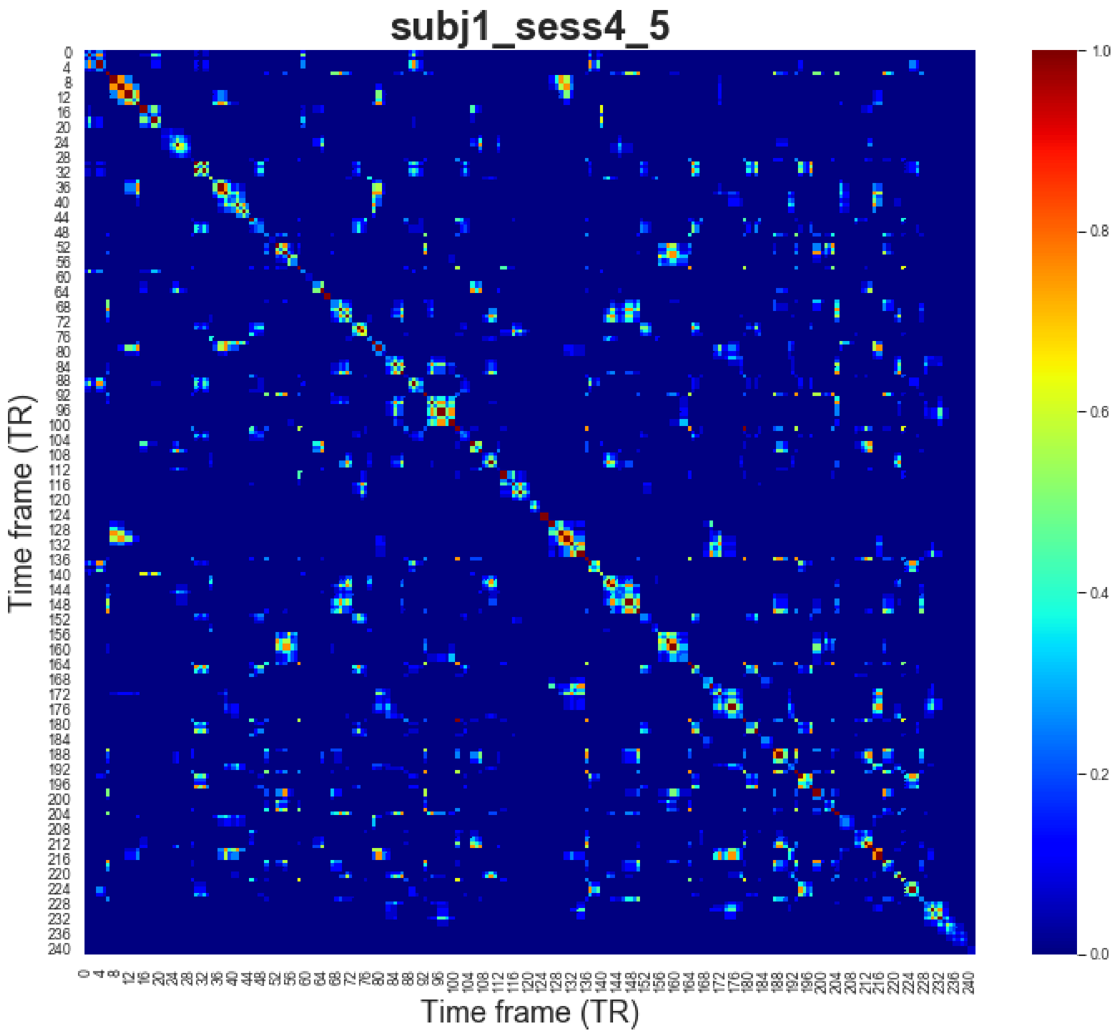

```
In [22]: sns.heatmap(TCMs.mean(axis=0), cbar=True, cmap='jet', figure=plt.figure(figsize=(12,1
         plt.title('{} (average)'.format(haxby.session_code), fontsize=24, fontweight='bold')
         plt.xlabel('Time frame (TR)', fontsize=18)
         plt.ylabel('Time frame (TR)', fontsize=18)
```

```
Out[22]: Text(86.7,0.5,'Time frame (TR)')
```

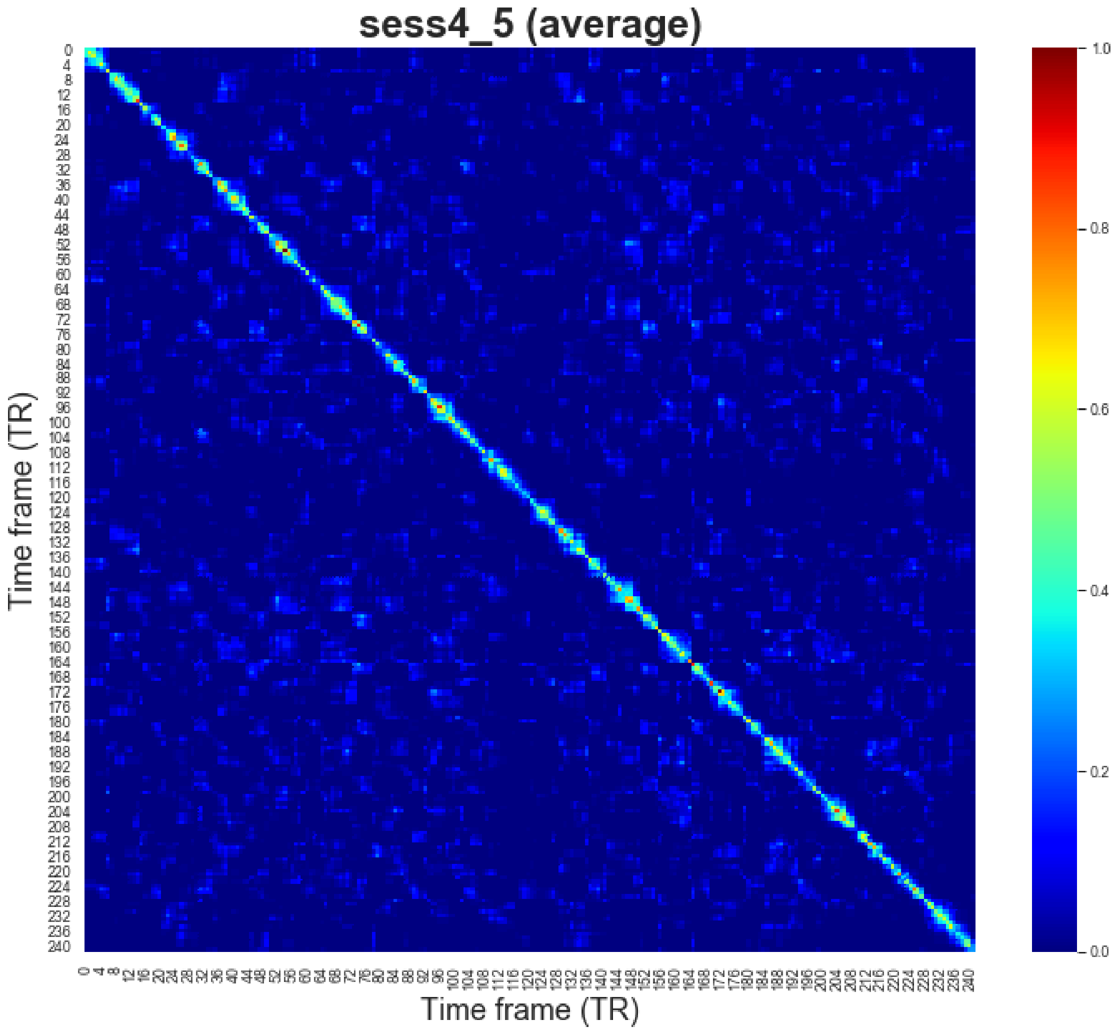

1.6.2 6.2 Visualize the normalized degree of nodes in the TCM over time

```
In [23]: ds.visuals.plot_temporal_degree(dG.TCM, title=subject.name)
```

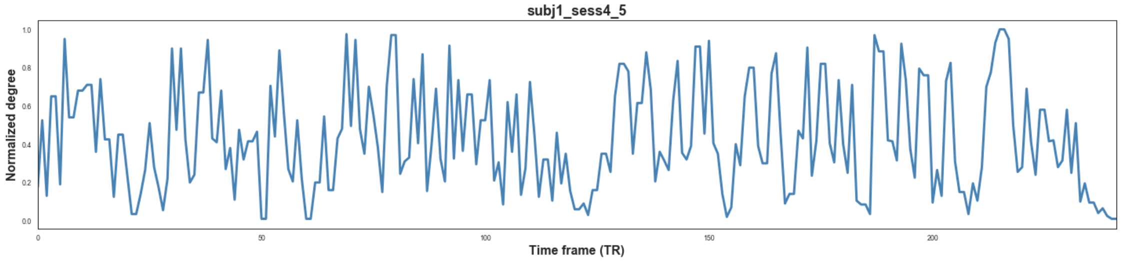


```
In [24]: ds.visuals.plot_temporal_degree(TCMs.mean(axis=0), title='{} (average)'.format(haxby.s
```

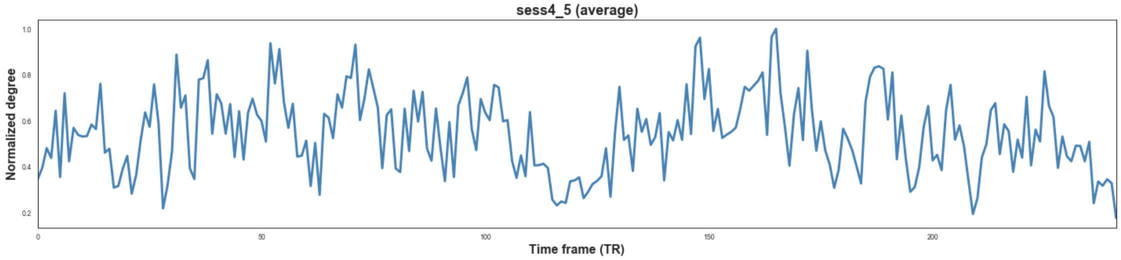

1.6.3 6.3 Visualize the degree of TCM for a single stimulus category

```
In [25]: # define subject, category
         subject = subjects[0]
         target_name = 'face'

         # mask target
         target = subject.target.copy().reset_index(drop=True)
         target = target.assign(data_id = target.index)
         meta = subject.meta.copy().reset_index(drop=True)

         # tcms
         tcms = []
         for session in [4, 5]:

             # define mask for each session
             target_mask = target[target_name].eq(1)
             session_mask = meta.session.eq(session)
             target_index = target[(target_mask & session_mask)].index.values
             target_bounds = np.min(target_index), np.max(target_index)

             # include 5 rest beforeblock
             rest_before = (target.loc[target['rest'].eq(1)]
                                  .data_id.ge(target_bounds[0]-5))
```

```
            rest_after = (target.loc[target['rest'].eq(1)]
                          .data_id.le(target_bounds[1]+5))
            rest_mask = (rest_before & rest_after)

            # mask targets for session, and rest before, after block
            target_mask = ((target_mask & session_mask) | (rest_mask & session_mask))
            target_mask_ix = np.ix_(target_mask, target_mask)
            trs = target_mask[target_mask].iloc[::].index.values

            # print some info
            print("Extracting TCM for category={}, session={}, onset={}, offset={}".format(
                target_name, session, trs[0]+5, trs[-1]-5
                 ))

            # extract tcm
            tcm = subject.mapped.dG.TCM.copy()
            tcm = tcm[target_mask_ix]

            # convert to dataframe, save
            df_tcm = pd.DataFrame(tcm, columns=trs, index=trs)
            tcms.append(df_tcm)


            # plot heatmap
            figure, axes = plt.subplots(1, 2, figsize=(20,8))
            ax = sns.heatmap(df_tcm, cbar=True, cmap='jet', ax=axes[0])
            ax = sns.heatmap(df_tcm>0, cbar=True, cmap='jet', axes=axes[1])
            for ax in axes:
                ax.set_title("category={}, session={}, onset={}, offset={}".format(
                    target_name, session, trs[0]+5, trs[-1]-5
                    ), fontsize=16)
            plt.suptitle(subject.name, fontsize=24, fontweight='bold')
            plt.show()


        # convert to array
        tcms = np.array([_.values for _ in tcms])
```

```
Extracting TCM for category=face, session=4, onset=49, offset=57
```

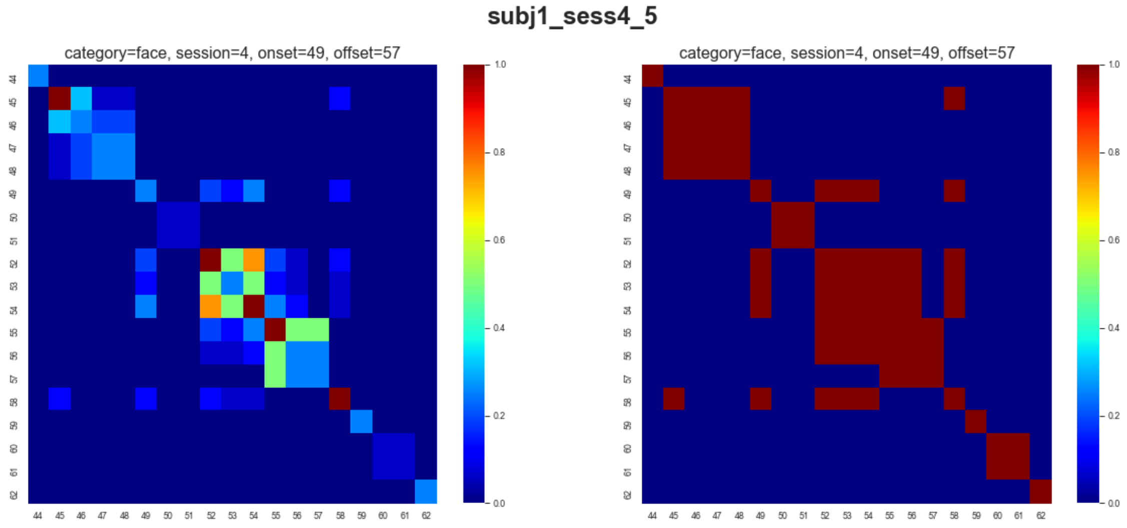


```
Extracting TCM for category=face, session=5, onset=156, offset=164
```

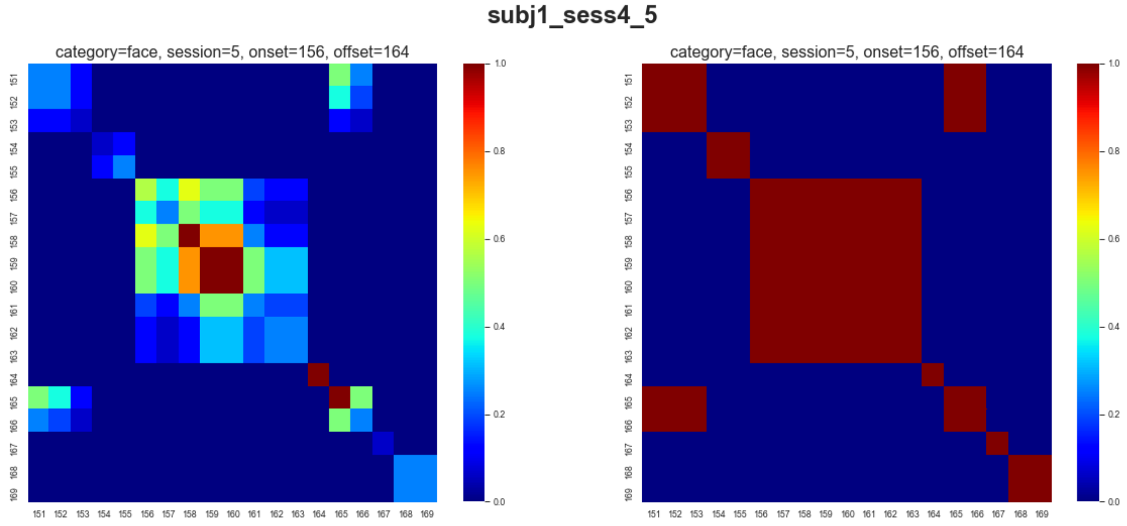


```
In [26]: # plot heatmap
         figure, axes = plt.subplots(1, 2, figsize=(20,8))
         ax = sns.heatmap(tcms.mean(0), cbar=True, cmap='jet', ax=axes[0])
         ax = sns.heatmap(tcms.mean(0)>0, cbar=True, cmap='jet', axes=axes[1])
         for ax in axes:
             ax.set_title("category={}, session={}, onset={}, offset={}".format(
                 target_name, session, trs[0]+5, trs[-1]-5
                 ), fontsize=16)
```

```
plt.suptitle(haxby.session_code, fontsize=24, fontweight='bold')
plt.show()
```

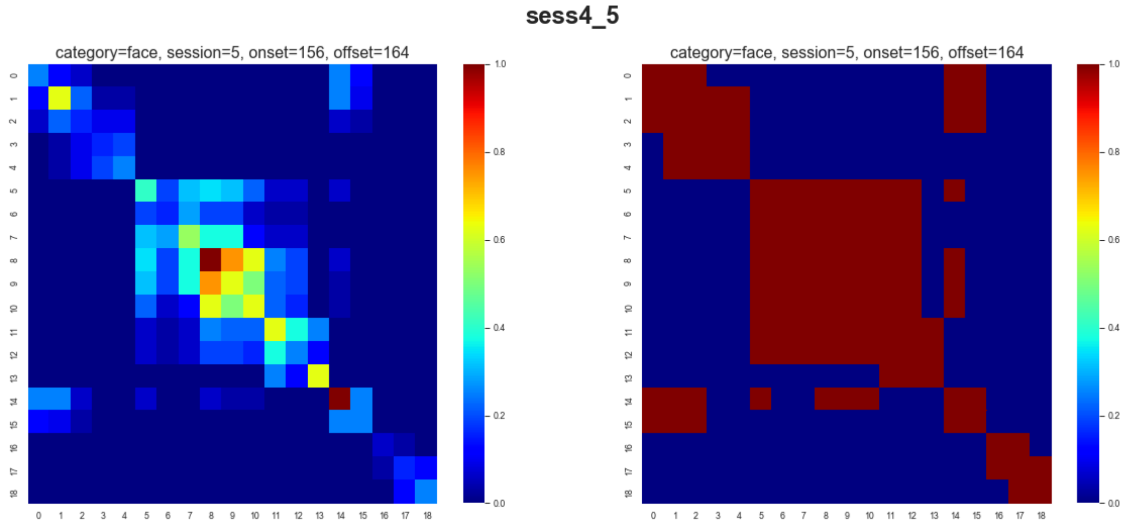

Supplement: Supplementary file 3 [file netn-03-763-suppData_2.pdf]
